# Supplementary material for: β-Globin Lentiviral Vectors Have Reduced Titers due to Incomplete Vector RNA Genomes and Lowered Virion Production
Source: Stem Cell Reports. 2020 Nov 12;16(1):198–211. doi: 10.1016/j.stemcr.2020.10.007 (PMC7897704; doi:10.1016/j.stemcr.2020.10.007)
Supplement: Document S1. Supplemental Experimental Procedures, Figures S1–S6, and Table S1 [file mmc1.pdf]

Stem Cell Reports, Volume *16*

Supplemental Information

β-Globin Lentiviral Vectors Have Reduced Titers due to Incomplete Vector RNA Genomes and Lowered Virion Production

Jiaying Han, Kevin Tam, Feiyang Ma, Curtis Tam, Bamidele Aleshe, Xiaoyan Wang, Jason P. Quintos, Marco Morselli, Matteo Pellegrini, Roger P. Hollis, and Donald B. Kohn

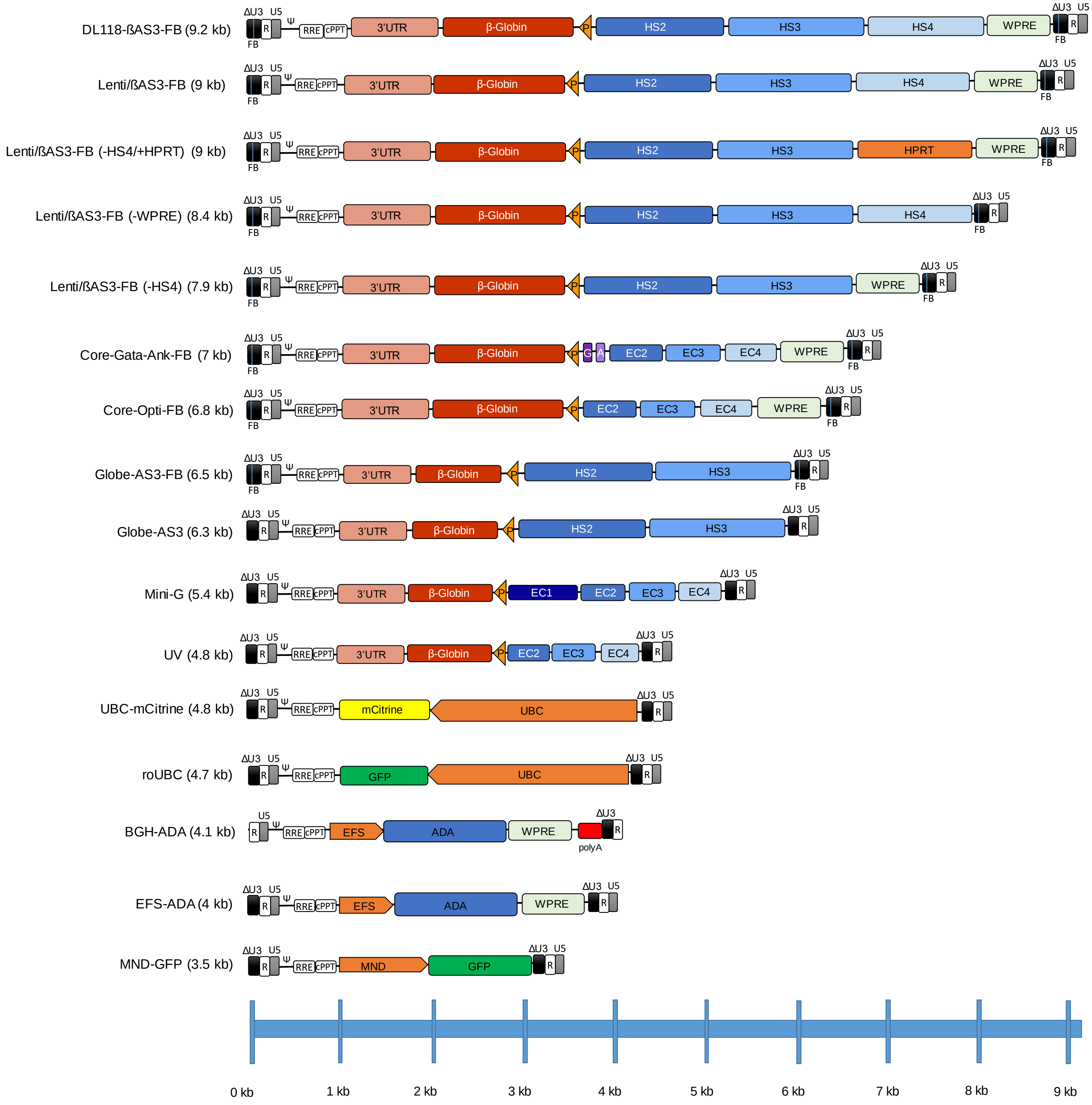
DL118-ßAS3-FB (9.2 kb)
Lenti/ßAS3-FB (9 kb)
Lenti/ßAS3-FB (-HS4/+HPRT) (9 kb)
Lenti/ßAS3-FB (-WPRE) (8.4 kb)
Lenti/ßAS3-FB (-HS4) (7.9 kb)
Core-Gata-Ank-FB (7 kb)
Core-Opti-FB (6.8 kb)
Globe-AS3-FB (6.5 kb)
Globe-AS3 (6.3 kb)
Mini-G (5.4 kb)
UV (4.8 kb)
UBC-mCitrine (4.8 kb)
roUBC (4.7 kb)
BGH-ADA (4.1 kb)
EFS-ADA (4 kb)
MND-GFP (3.5 kb)
ΔU3
U5
R
FB
Ψ
RRE
cPPT
3'UTR
β-Globin
P
HS2
HS3
HS4
WPRE
HPRT
G
A
EC1
EC2
EC3
EC4
mCitrine
UBC
GFP
EFS
ADA
polyA
MND
0 kb
1 kb
2 kb
3 kb
4 kb
5 kb
6 kb
7 kb
8 kb
9 kb

Figure S1. Maps of the lentiviral vector proviruses. All vectors consist of a pCCL backbone and differ only by the internal promoters and transgene cassettes. ΔU3, R, U5 are long terminal repeats (LTR). FB, FII-BEAD insulator. Ψ, packaging signal. RRE, Rev response element. cPPT, central polypurine tract. 3' UTR, ß-globin gene 3' UTR enhancer elements. P, promoter. G, Gata1 erythroid transcription factor binding site. A, ankyrin-1 barrier insulator element. HS2, HS3, HS4, ß-globin locus hypersensitive sites 2, 3 and 4. EC2, EC3, EC4, enhancer core elements. WPRE, mutated woodchuck hepatitis virus post-transcriptional regulatory element. ß-Globin, ß-Globin gene cassette. UBC, ubiquitin C promoter. EFS, elongation factor 1 alpha promoter. ADA, codon-optimized adenosine deaminase cDNA. BGH-ADA is shown in its RNA form because it is a truncated vector that cannot integrate.

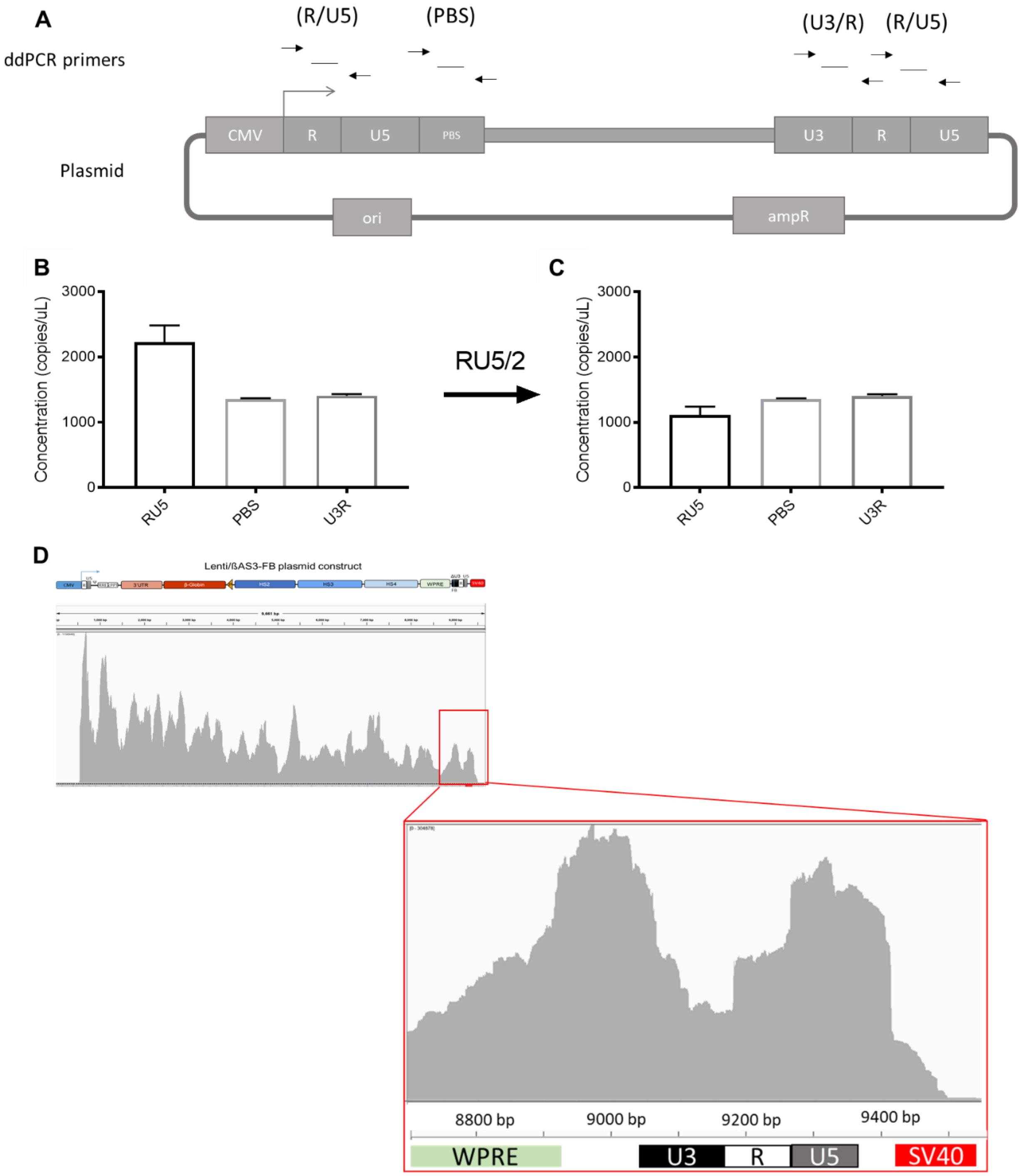

A
ddPCR primers
(R/U5)
(PBS)
(U3/R)
(R/U5)
CMV
R
U5
PBS
U3
R
U5
Plasmid
ori
ampR
B
Concentration (copies/uL)
3000
2000
1000
0
RU5
PBS
U3R
RU5/2
C
Concentration (copies/uL)
3000
2000
1000
0
RU5
PBS
U3R
D
Lenti/ßAS3-FB plasmid construct
8800 bp
9000 bp
9200 bp
9400 bp
WPRE
U3
R
U5
SV40

Figure S2. Quantification of ddPCR primer amplification efficiencies and viral RNA transcription readthrough. (A) Schematic representation of primer binding sites in vector plasmids. (B) Copy number/ul by R/U5, PBS and U3/R primers and probes in Lenti/ßAS3-FB plasmids (n=2 independent experiments). (C) Normalized copy number/ul. R/U5 copy number was divided by two, because plasmids contain two copies of R/U5 (bars represent mean with SEM; n=2 independent experiments). (D) Alignment of RNA-seq reads to 3'LTR to SV40 polyadenylation sites in Integrative Genomics Viewers.

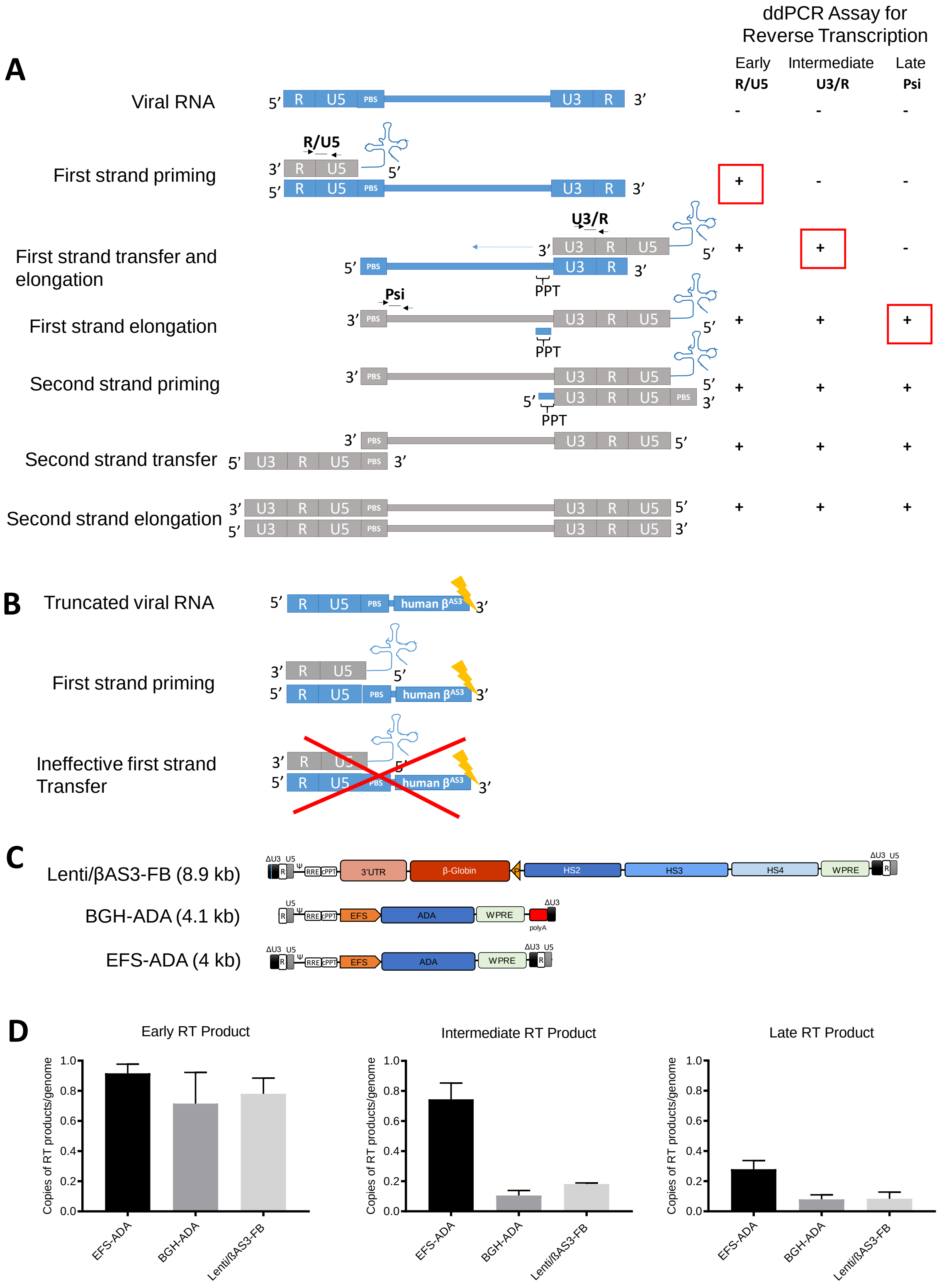

ddPCR Assay for Reverse Transcription
Early R/U5
Intermediate U3/R
Late Psi
A
Viral RNA
First strand priming
First strand transfer and elongation
First strand elongation
Second strand priming
Second strand transfer
Second strand elongation
R/U5
U3/R
Psi
PPT
PBS
B
Truncated viral RNA
First strand priming
Ineffective first strand Transfer
human β^AS3
C
Lenti/βAS3-FB (8.9 kb)
BGH-ADA (4.1 kb)
EFS-ADA (4 kb)
3'UTR
β-Globin
HS2
HS3
HS4
WPRE
EFS
ADA
polyA
D
Early RT Product
Intermediate RT Product
Late RT Product
Copies of RT products/genome
EFS-ADA
BGH-ADA
Lenti/βAS3-FB

Figure S3. Lenti/ßAS3-FB failed reverse transcription at the first strand transfer step. (A) Schematic representation of the mechanism of reverse transcription (RT) and the ddPCR assay used to quantify reverse transcribed products. The ddPCR primers and probes were designed to quantify different stages of RT when RT products become detectable. The R/U5 primers quantify initial reverse transcribed products, the U3/R primers quantify intermediate reverse transcribed products, and the Psi primers quantify late reverse transcribed products. Blue box: viral RNA. Gray box: viral DNA. (B) Proposed mechanism of truncated RNA failing reverse transcription at the first strand transfer step. (C) Schematic representation of the vector RNA of EFSADA and the truncated version BGHADA. (D) Quantification of reverse transcribed products of Lenti/ßAS3-FB, EFS-ADA and BGH-ADA LVs in KG1a cells (bars represent mean with SEM; n=5 dishes of identical cultures from 2 independent experiments). BGH-ADA is a truncated form of EFSADA LV with the Bovine Growth Hormone (BGH) polyadenylation signal between WPRE and 3'LTR. KG1a cells were transduced with Lenti/ßAS3-FB, EFS-ADA, or BGH-ADA LVs at equal levels of Initial RNA in the presence of PGE2, poloxamer, and benzonase. Cells were harvested at 24 h for gDNA extraction. Reverse transcription products were quantified by ddPCR with R/U5, U3/R, Psi, and SDC4 primers.

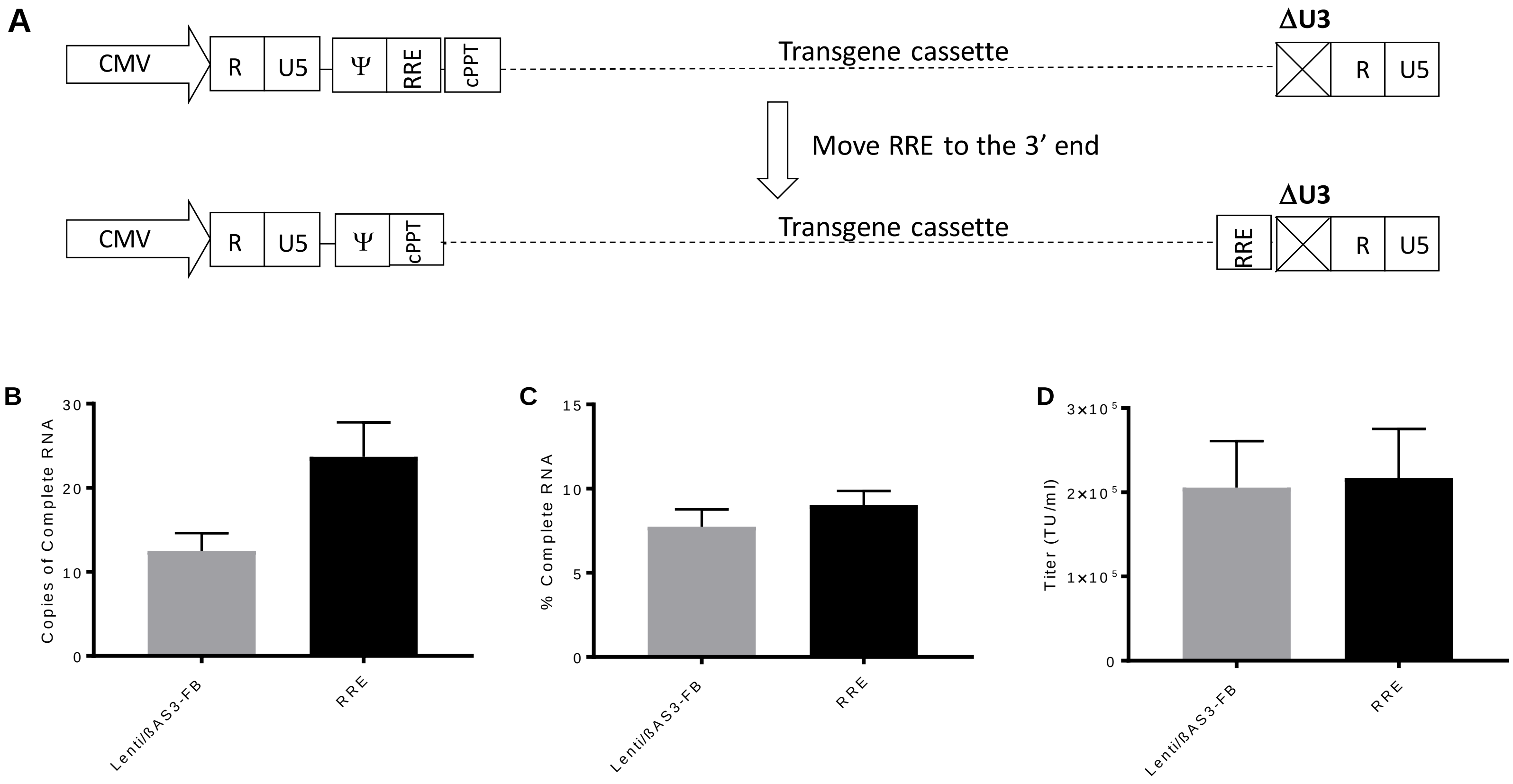
A
CMV
R
U5
Ψ
RRE
cPPT
Transgene cassette
ΔU3
R
U5
Move RRE to the 3' end
CMV
R
U5
Ψ
cPPT
Transgene cassette
RRE
ΔU3
R
U5
B
Copies of Complete RNA
30
20
10
0
Lenti/ßAS3-FB
RRE
C
% Complete RNA
15
10
5
0
Lenti/ßAS3-FB
RRE
D
Titer (TU/ml)
3×10^5
2×10^5
1×10^5
0
Lenti/ßAS3-FB
RRE

Figure S4. Moving the RRE element to the 3' LTR did not increase viral titer. (A) Schematic representation of the vector constructs. The RRE element in Lenti/ßAS3-FB was moved from a site downstream of the 5' LTR to a site upstream of the 3' LTR to generate a new vector termed as Lenti/ßAS3-FB-RRE. (B) Absolute quantification and (C) percentage of viral RNA from unconcentrated viral supernatant of Lenti/ßAS3-FB-RRE and Lenti/ßAS3-FB measured by ddPCR with R/U5 and U3/R primers and probes (n=3 dishes of identical cultures treated and analyzed in two independent experiments). (D) Viral titers of Lenti/ßAS3-FB-RRE and Lenti/ßAS3-FB LVs (n=3 dishes of identical cultures treated and analyzed in two independent experiments; bars represent mean with SEM).

.

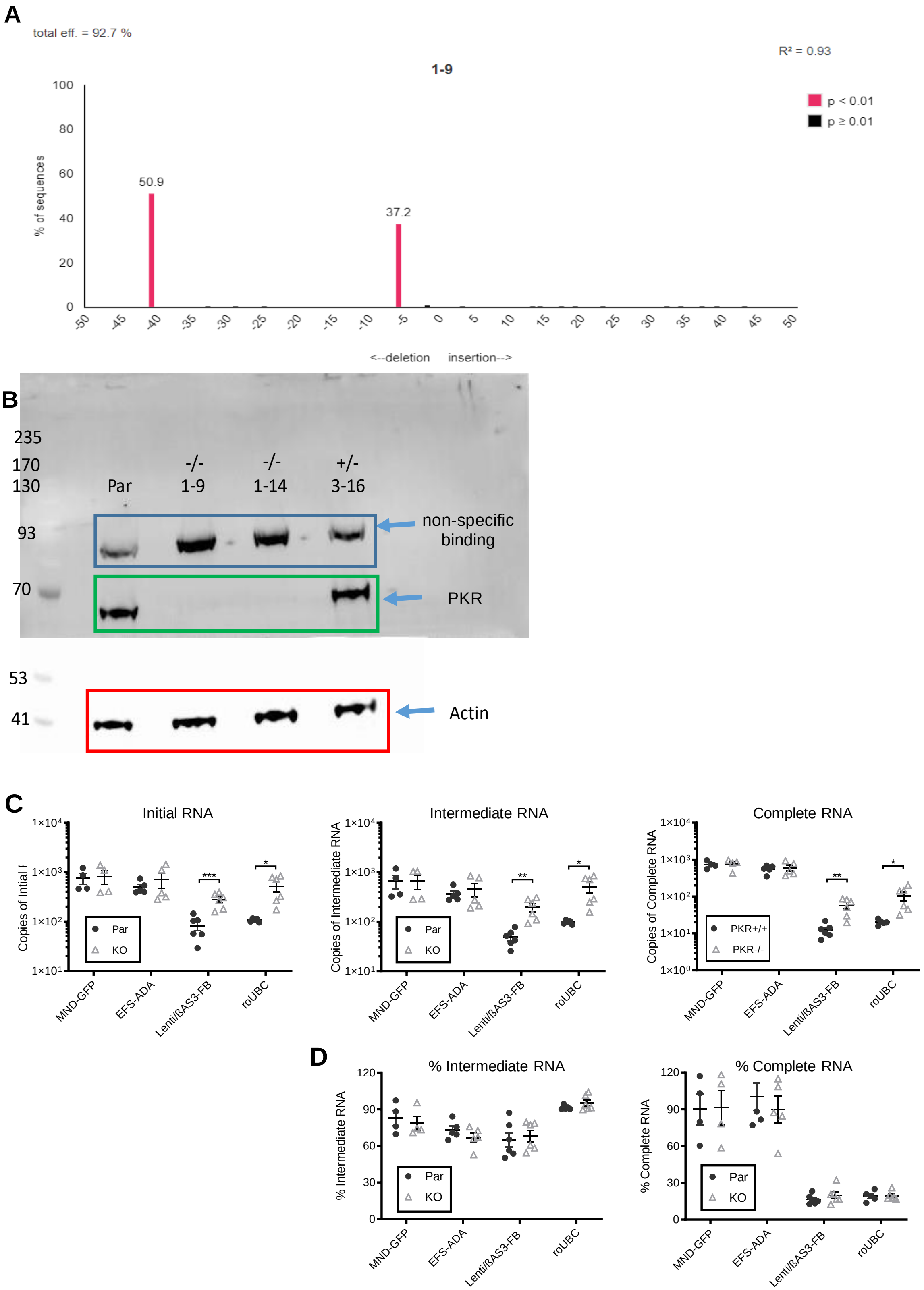
A
total eff. = 92.7 %
R² = 0.93
1-9
p < 0.01
p ≥ 0.01
% of sequences
50.9
37.2
<--deletion insertion-->
B
235
170
130
93
70
53
41
Par
-/- 1-9
-/- 1-14
+/- 3-16
non-specific binding
PKR
Actin
C
Initial RNA
Intermediate RNA
Complete RNA
Copies of Intermediate RNA
Copies of Complete RNA
Par
KO
PKR+/+
PKR-/-
MND-GFP
EFS-ADA
Lenti/ßAS3-FB
roUBC
D
% Intermediate RNA
% Complete RNA

Figure S5. Knocking out *PKR* increased the level of all three RNA species in reverse-oriented vectors without changing the RNA completeness. (A) Assessment of genome disruption by TIDE sequencing of the *PKR-/-* isogenic 293T cell line. Cas9 and guide RNA were introduced to the cells by lipofection of PX330 plasmids bearing the Cas9 and guide RNA sequence targeting PKR. Cells were sorted for single cell clones, and the isogenic cell clones were harvested three weeks later for genomic DNA extraction and Tide sequencing analysis. (B) PKR protein expression in different isogenic cell clones measured by Western blot. Clones 1-9 and 1-14 were homozygous *PKR-/-* cell clones, and 3-16 was a heterozygous *PKR+/-* cell clone by TIDE sequencing. Parental (Par) 293T cell lysate was used as a positive control. ß-actin was used as the loading control. The expected size of PKR protein was 74 kDa. (C) Absolute quantification of Initial (R/U5), Intermediate (PBS), and Complete (U3/R) RNA in vector particles by ddPCR (bars represent mean with SEM; n=4-6 dishes of identical cultures treated and analyzed in 3 independent experiments; unpaired t test, *p<0.05, **p<0.01, ***p<0.001). (D) Percentage Intermediate and Complete RNA in vector particles measured by ddPCR (bars represent mean with SEM; n=4-6 dishes of identical cultures from 3 independent experiments; unpaired t test, *p<0.05, **p<0.01, ***p<0.001). RNA was extracted from vector particles, treated with DNAse, reverse transcribed with random primers, and quantified by ddPCR.

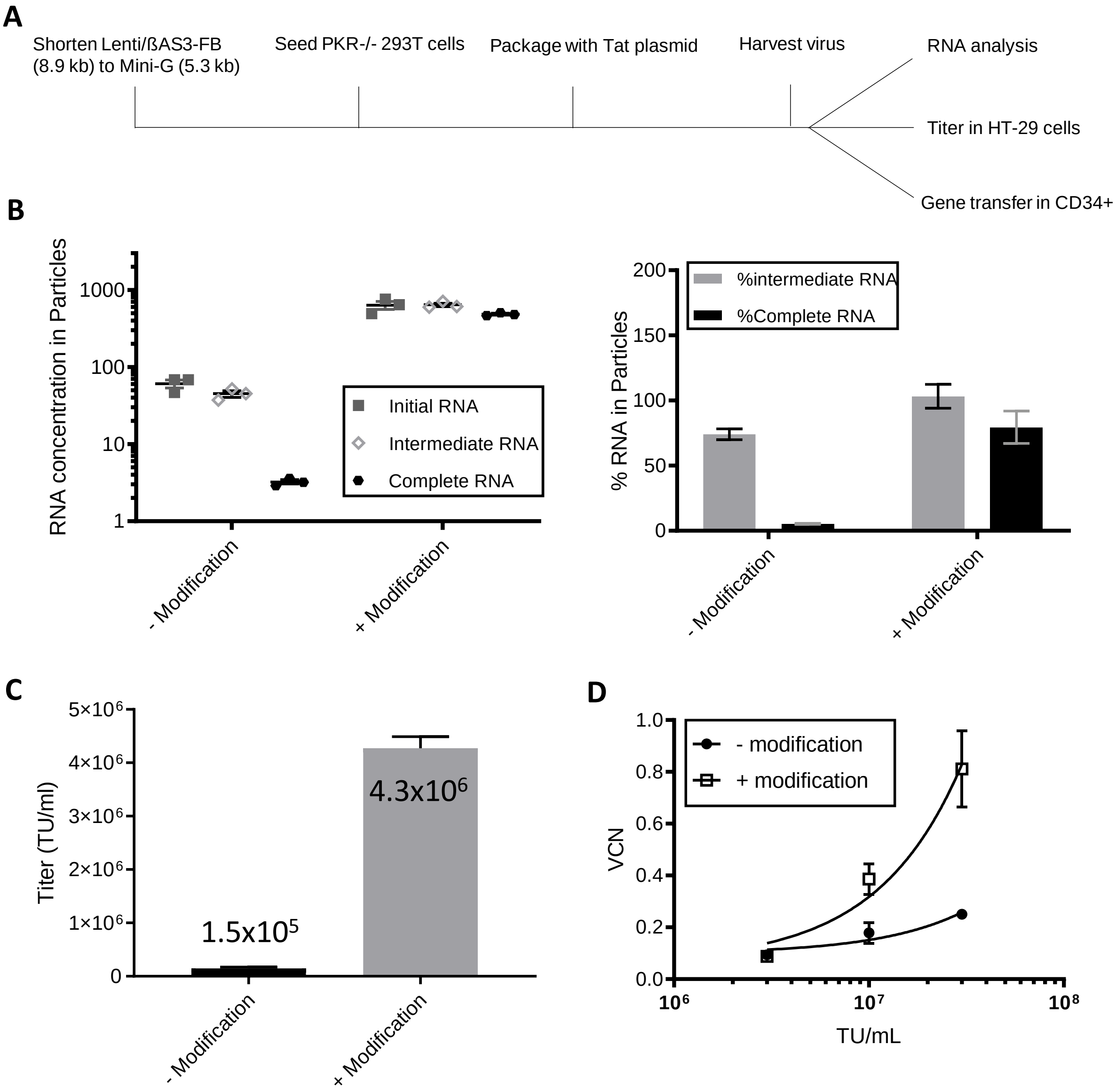
A
Shorten Lenti/ßAS3-FB
(8.9 kb) to Mini-G (5.3 kb)
Seed PKR-/- 293T cells
Package with Tat plasmid
Harvest virus
RNA analysis
Titer in HT-29 cells
Gene transfer in CD34+
B
RNA concentration in Particles
1000
100
10
1
Initial RNA
Intermediate RNA
Complete RNA
- Modification
+ Modification
% RNA in Particles
200
150
100
50
0
%intermediate RNA
%Complete RNA
- Modification
+ Modification
C
Titer (TU/ml)
5×10^6
4×10^6
3×10^6
2×10^6
1×10^6
0
1.5x10^5
4.3x10^6
- Modification
+ Modification
D
VCN
1.0
0.8
0.6
0.4
0.2
0.0
- modification
+ modification
10^6
10^7
10^8
TU/mL

Figure S6. Combining the three modifications for the production of ß-globin vectors increased viral RNA, vector titer and CD34+ cell infectivity. (A) The schematic representation of the three modifications: shortening the vector length, packaging with TAT plasmids and packaging in PKR-/- cells. (B) The absolute quantification and percentage of viral RNA in viral particles measured by ddPCR. (C) Titers of unconcentrated ß-globin vectors +/- 3 modifications (bars represent mean with SEM; n=3 dishes of identical cultures treated and analyzed in 2 independent experiments). (D) Infectivity of ß-globin vectors +/- 3 modifications in BM CD34+ HSPCs (bars represent mean with SEM; n=3 independent experiments; linear regression, comparison of the slopes, p=0.0009). Data represent measurements from three independent human BM CD34+ HSPCs donors.

Table S1. Sequences of primer and probes

Name	Sequence 5'→3'
R/U5 FWD	GCTAACTAGGGAACCCACTGCT
R/U5 REV	GGGTCTGAGGGATCTCTAGTTACCA
R/U5 Probe	FAM- CTTCAAGTAGTGTGTGCCCGTCTGT-31ABFQ
PBS FWD	AAGTAGTGTGTGCCCGTCTG
PBS REV	CCTCTGGTTTCCCTTTCGCT
PBS Probe	FAM-CCCTCAGACCCTTTTAGTCAGTGTGGAAAATCTCTAG-31ABFQ
U3/R FWD	AGCAGTGGGTTCCCTAGTTAG
U3/R REV	GGGACTGGAAGGGCTAATTC
U3/R Probe	FAM-AGAGACCCAGTACAAGCAAAAAGCAG-31ABFQ
Psi FWD	CTTGAAAGCGAAAGGGAAACC
Psi REV	CGCACCCATCTCTCTCCTTCT
Psi Probe	FAM-AGCTCTCTCGACGCAGGACTCGGC-31ABFQ
SDC4 FWD	CAGGGTCTGGGAGCCAAGT
SDC4 REV	GCACAGTGCTGGACATTGACA
SDC4 Probe	FAM - CCCACCGAACCCAAGAAACTAGAGGAGAAT - TAMRA

Supplemental Methods

Reverse Transcription Assay

Raw viral supernatants of EFS-ADA, Lenti/βAS3-FB and BGH-ADA vectors with the same amounts of Initial RNA were used to transduce KG1a cells. Benzonase, 50 U/mL (MilliporeSigma, Burlington, MA) and transduction enhancers, Poloxamer 1 mg/mL (Kolliphor P338; BSAF, Ludwigshafen, Germany) and PGE2 10 µM (Cayman Chemicals, Ann Arbor, MI, USA) were added to the cells at the same time as the addition of LV. The preparation of transduction enhancers was previously described in Masiuk et al (Masiuk et al., 2019). Cells were harvested at 24 hours after transduction, and DNA was extracted using Qiagen Blood and Cell culture DNA Mini Kit (Qiagen).

The copies of reverse-transcribed DNA products were measured by ddPCR, calculated as the ratio of the copies of the viral genomes to the copies of the *SDC4* endogenous reference gene. Different PCR primers were used to quantify early, intermediate and late reverse transcribed products. The R/U5 primers and probe were used to quantify early reverse transcribed products, and the U3/R primer and probe were used to quantify intermediate reverse transcribed products, as described earlier. The cycling conditions for ddPCR consisted of 95°C for 10 min for one cycle, (94°C for 30 s and 60°C for 1 min) for 40 cycles, 10 min at 98°C for one cycle, and a 12°C hold.

Generation of *PKR-/-* 293T Cell Line

Single guide RNA (sgRNA) targeting *PKR* exon 1 were designed using Benchling CRISPR online tool, and paired oligonucleotides were ordered from Integrated DNA Technologies (San Diego, CA). Paired oligonucleotides encoding the sgRNA were annealed and cloned into the PX330-U6-Chimeric_BB-CBh-hSpCas9 expression vector (#42230, Addgene, Watertown, Massachusetts) as previously described (Hsu et al., 2013; Cong et al., 2013). Maxi-prepped expression plasmids were filtered through a 0.22 µM filter and introduced to 293T cells by transient transfection with 5 µg of plasmids, 1.5 mL OPTI-MEN (ThermoFisher, Waltham, MA) and 35 uL TransIT-293 (Mirus Bio) in a 10 cm plate, following the manufacturer's protocol. Cell sorting to isolate single cells was conducted 48 hours after transfection at The UCLA Broad Stem Cell

Research Center Flow Cytometry Core. After three weeks of culture, isogenic cell clones were collected for genomic DNA extraction, PCR amplification, Tide sequencing analysis, and Western blot analysis for PKR protein expression, as previously described in Hu et al to isolate *PKR*-/- clones with bi-allelic *PKR* knock-out and no detectable residual PKR expression (Birkman et al., 2014; Hu et al, 2018),. One *PKR* -/- clone was expanded and used for LV packaging.

LV Production & Titration

LVs were packaged by transient transfection of 293T cells with fixed amounts of HIV Gag/Pol, Rev and VSV-G expression plasmids and equimolar amounts of each of the different vector transfer plasmids using TransIT-293 (Mirus Bio, Madison, WI). Approximately 20 hours after transfection, the culture medium was changed to D10 containing 10 mM sodium butyrate and 20 mM HEPES. After 6-8 hours, cells were washed with PBS and cultured in fresh D10. Viral supernatants were collected ~40 hours after sodium butyrate induction and filtered through a 0.45-µm filter. Viral supernatants were processed and concentrated by ultracentrifugation at 26,000 rpm for 90 minutes, when necessary. Both unconcentrated and concentrated viral supernatant were harvested and stored at -80°C.

Viral supernatant was titrated in HT29 cells as described in the method section. VCN was determined as the ratio of the copies of the HIV-1 PSI region to the copies of the *SDC4* endogenous reference gene. Droplet generation was performed as described in Hindson et al (Hindson et al., 2011). The droplet emulsion was then transferred to a 96-well polypropylene plate (Eppendorf, Hamburg, Germany) and amplified in a conventional thermal cycler (T100; Bio-Rad). Thermal cycling conditions consisted of 95°C for 10 min for one cycle, (94°C for 30 s and 60°C for 1 min) for 55 cycles, 10 min at 98°C for one cycle, and a 12°C hold. After PCR, the 96-well plate was transferred to a ddPCR droplet reader (Bio-Rad) for data acquisition and analysis performed with the QuantaSoft software (Bio-Rad).

Supplemental References

Brinkman, E. K., Chen, T., Amendola, M. & Steensel, B. Van. Easy quantitative assessment of genome editing by sequence trace decomposition. 42, (2014).

Hindson, B. J. *et al.* High-Throughput Droplet Digital PCR System for Absolute Quantitation of DNA Copy Number. 8604–8610 (2011).

Hsu, P. D. *et al.* DNA targeting specificity of RNA-guided Cas9 nucleases. 31, (2013).

Cong, L. *et al.* Multiplex genome engineering using CRISPR/Cas systems. *Science.* 339, 819–823 (2013).

Hu, P. *et al.* Superior lentiviral vectors designed for BSL-0 environment abolish vector mobilization. *Gene Ther.* 454–472 (2018). doi:10.1038/s41434-018-0039-2

Masiuk, K. E. *et al.* PGE2 and Poloxamer Synperonic F108 Enhance Transduction of Human HSPCs with a β-Globin Lentiviral Vector. *Mol. Ther. Methods Clin. Dev.* 13, 390–398 (2019).
