# Supplementary material for: β-Globin Lentiviral Vectors Have Reduced Titers due to Incomplete Vector RNA Genomes and Lowered Virion Production
Source: Stem Cell Reports. 2020 Nov 12;16(1):198–211. doi: 10.1016/j.stemcr.2020.10.007 (PMC7897704; doi:10.1016/j.stemcr.2020.10.007)
Supplement: Document S2. Article plus Supplemental Informatio [file mmc2.pdf]

Stem Cell Reports
Article
OPEN ACCESS

β-Globin Lentiviral Vectors Have Reduced Titers due to Incomplete Vector RNA Genomes and Lowered Virion Production

Jiaying Han,[1] Kevin Tam,[2] Feiyang Ma,[2] Curtis Tam,[2] Bamidele Aleshe,[4] Xiaoyan Wang,[3] Jason P. Quintos,[4] Marco Morselli,[2] Matteo Pellegrini,[2] Roger P. Hollis,[4] and Donald B. Kohn[1,4,5,6,7,*]
[1]Department of Molecular and Medical Pharmacology, David Geffen School of Medicine, University of California, Terasaki Life Sciences Building, 610 Charles E. Young Drive East, Los Angeles, CA 90095-1489, USA
[2]Department of Molecular, Cell, and Developmental Biology, University of California, Los Angeles, USA
[3]Department of General Internal Medicine and Health Services Research, UCLA, Los Angeles, CA, USA
[4]Department of Microbiology, Immunology & Molecular Genetics, David Geffen School of Medicine, University of California, Los Angeles, USA
[5]Department of Pediatrics, David Geffen School of Medicine, University of California, Los Angeles, USA
[6]The Eli & Edythe Broad Center of Regenerative Medicine & Stem Cell Research, University of California, Los Angeles, USA
[7]UCLA Jonsson Comprehensive Cancer Center, Los Angeles, USA
*Correspondence: dkohn1@mednet.ucla.edu
https://doi.org/10.1016/j.stemcr.2020.10.007

SUMMARY

Lentiviral vectors (LVs) commonly used for the treatment of hemoglobinopathies often have low titers and sub-optimal gene transfer efficiency for human hematopoietic stem and progenitor cells (HSPCs), hindering clinical translation and commercialization for *ex vivo* gene therapy. We observed that a high percentage of β-globin LV viral genomic RNAs were incomplete toward the 3′ end in packaging cells and in released vector particles. The incomplete vector genomes impeded reverse transcription in target cells, limiting stable gene transfer to HSPCs. By combining three modifications to vector design and production (shortening the vector length to 5.3 kb; expressing HIV-1 Tat protein during packaging; and packaging in *PKR*−/− cells) there was a 30-fold increase in vector titer and a 3-fold increase in vector infectivity in HSPCs. These approaches may improve the manufacturing of β-globin and other complex LVs for enhanced gene delivery and may facilitate clinical applications.

INTRODUCTION

In multiple clinical trials, autologous hematopoietic stem cell (HSC) transplantation in combination with gene therapy has produced clinical benefits with desirable long-term engraftment of gene-corrected HSCs and stable gene expression (Aiuti et al., 2009; Biffi et al., 2011; Cartier et al., 2012; De Ravin et al., 2016). Despite these early successes, the lengthy and complex nature of some lentiviral vectors (LVs) has led to several challenges for clinical translation. We and others observed that titers of LVs decrease substantially with increasing proviral length (Kumar et al., 2001; Morgan et al., 2019). The reduction in titer, especially in the vectors with complex expression cassettes, creates a barrier for scaling up good manufacturing practices-grade vector production and significantly increases the production cost for clinical trials and commercialization (Morgan et al., 2017). In addition, even when adjusted to matching transduction units (TUs), the complex vectors do not transduce hematopoietic stem and progenitor cells (HSPCs), which are relatively resistant to LV infection, as efficiently as the simple vectors, often failing to achieve high vector copy number (VCN) and transgene expression to provide therapeutic benefits (Kanter et al., 2017). Low titer and infectivity associated with complex LVs present hurdles to the use of these LVs to treat diseases with complex transgene cassettes, such as Duchenne muscular dystrophy (15-kb proviral length) and sickle cell disease (SCD) (~9-kb proviral length) (Counsell et al., 2017; Romero et al., 2013).

SCD is one of the most common monogenic disorders worldwide, with >250,000 new patients each year (Hoffman, 2018; Modell and Darlison, 2008). SCD is caused by a point mutation in the sixth codon of the β-globin gene, resulting in an abnormal hemoglobin molecule that aggregates at low oxygen tension, leading to rigid sickle-shaped red blood cells that occlude small blood vessels (Harris, 1950; Nash et al., 1986; Pauling et al., 1949). Using a dominant anti-sickling β-globin gene (e.g., T87Q or βAS3-globin) integrated into the patient's own HSCs, which are then reinfused back to the patient, can potentially provide healthy non-sickling erythrocytes to patients throughout life. However, the development of β-globin LVs that allows efficient gene transfer into HSPCs while maintaining stable, high-level, and lineage-specific expression of the β-globin gene has been historically challenging (Lisowski and Sadelain, 2008). Many regulatory elements from the β-globin locus, such as 5′ and 3′ untranslated regions, introns, and parts of the β-globin locus control region (LCR), are necessary to maintain stable, high-level expression of the therapeutic gene. In addition, the β-globin gene cassette is typically placed in the anti-sense orientation relative to the direction of genome transcription during packaging, to prevent splicing of introns in the

This is an open access article under the CC BY license (http://creativecommons.org/licenses/by/4.0/).

transgene. The complex nature of the β-globin LV, namely the long genome length and reverse orientation of the β-globin cassette, lowers the viral titer and infectivity.

Small-molecule transduction enhancers have been reported to improve the transduction efficiency of HSPCs (Heffner et al., 2018; Masiuk et al., 2019; Petrillo et al., 2018). Nonetheless, transduction enhancers have no effect on the intrinsic infectious properties of the LVs, such as viral RNA transcription and infectious particle production. New methods to increase titer and gene transfer that can be used in combination with transduction enhancers will lead to additional clinical and commercial benefits.

Although the main reason for poor infectivity and low titer has been attributed to the increased proviral length, the effects of complex proviral construct on lentiviral life cycle have not been fully explored. Therefore, a systematic study of the lentiviral life cycle was conducted to compare a "well-behaved" simple vector, EFS-ADA (4 kb), and a "poorly performing" complex β-globin vector, Lenti/βAS3-FB (8.9 kb). Five steps of the lentiviral life cycle were examined: (1) viral RNA production in packaging cells, (2) viral RNA incorporation into virions, (3) physical vector particle formation, (4) reverse transcription, and (5) integration.

Our results showed that the viral RNAs of long complex vectors in packaging cells were predominantly incomplete at the 3′ end and failed reverse transcription in target cells at the first-strand transfer step, thus reducing the amount of vector DNA available for integration. Shortening the vector length and packaging with HIV-1 Tat expression plasmids improved the viral RNA production for the complex vectors. In addition, we observed that the Lenti/βAS3 vector with its β-globin gene expression unit in reverse orientation yielded fewer virion particles (measured by p24 Gag) than were produced with forward-oriented vectors and even with empty vectors lacking any genome. Knocking out protein kinase R (PKR) in HEK293T packaging cells increased virion production with a proportionate increase in virion RNA released into medium, improving titer and infectivity. These findings may be applied for clinical gene therapy not only with β-globin vectors, but also with many complex vectors with low titer and infectivity.

RESULTS

Complex Vectors Had Low Titer and Deficient Gene Transfer

Lenti/βAS3-FB is an 8.9-kb LV carrying a complex anti-sickling β-globin gene cassette, as described previously by Romero et al. (2013). The β-globin gene is driven by the endogenous β-globin promoter and enhancers in the anti-sense (reverse) orientation, with the expression regulated by a "mini" LCR comprising portions of the β-globin locus DNase hypersensitive sites (HSs) HS2, HS3, and HS4, as shown in Figure 1A. In addition, Lenti/βAS3-FB contains the woodchuck hepatitis virus post-transcriptional regulatory element (wPRE) and a 77-bp insulator in the U3 region of the 3′ long terminal repeat (LTR), termed as FB (FII-BEAD). EFS-ADA is a 4-kb LV for treating ADA severe combined immunodeficiency (ADA-SCID) that is capable of high-level and consistent *ADA* gene transfer and expression (Carbonaro et al., 2014; Schambach et al., 2006). The EFS-ADA vector consists of the EFS promoter driving the expression of a codon-optimized human *ADA* gene cassette followed by the wPRE, all in the sense (forward) orientation. Both vectors consist of a pCCL backbone and differ only by the internal promoters and transgene cassettes (Zufferey et al., 1998). We defined Lenti/βAS3-FB as a complex LV because of the lengthy viral genome, additional enhancer elements for lineage-specific expression, and anti-sense orientation of the transgene cassette.

To evaluate the viral titer of the Lenti/βAS3-FB LV in comparison with the EFS-ADA vector, LVs were packaged concurrently in 293T cells, and the titers of the unconcentrated viral supernatants were tested in parallel with the HT-29 cell line. The titer of Lenti/βAS3-FB was ~12-fold lower than the titer of EFS-ADA ($p < 0.001$, Wilcoxon rank-sum test) (Figure 1B).

To characterize the gene transfer efficiency of these vectors in clinically relevant target cells, prestimulated human bone marrow (BM) CD34+ HSPCs from healthy donors were transduced at three doses of TUs, and the integrated VCN were measured by ddPCR 12 days after transduction. Lenti/βAS3-FB displayed less-efficient gene transfer to human BM CD34+ cells than EFS-ADA (comparison of the slopes by linear regression, $p = 0.006$), especially at higher doses (Figure 1C). At the highest dose tested, 1×10^8 TU/mL, the average VCN of Lenti/βAS3-FB was 0.88 ± 0.23 versus 3.51 ± 0.83 for EFS-ADA in three independent donors, resulting in a 4-fold difference in transduction between the two LVs despite using the same MOIs. We subsequently used Lenti/βAS3-FB and EFS-ADA as examples of complex and simple LVs to study different steps of the lentiviral life cycle, identify the blocks that restrict the performance of complex vectors, and develop strategies to improve the titer and infectivity of complex vectors.

Viral RNA of the Complex Vectors Was Truncated in Packaging Cells and in Vector Particles

To quantify RNA species at different stages of transcription, we designed multiple ddPCR primers and probes along the viral RNA. Because transcription of vector plasmids starts at the 5′ LTR driven by the CMV promoter, RNA is transcribed in the following order: R/U5, primer binding site (PBS), transgene, and U3/R. R/U5 primers and probe were used

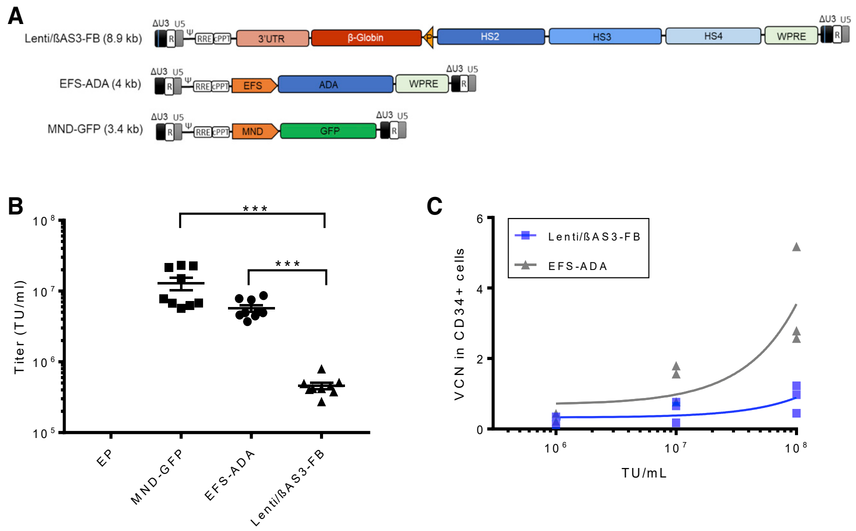

Figure 1. Complex β-Globin Vector, Lenti/βAS3-FB, Had Low Titer and Infectivity in Human Bone Marrow CD34+ HSPCs

(A) Maps of the proviral forms of EFS-ADA and Lenti/βAS3-FB LVs. Both vectors consist of a pCCL backbone and differ only by the internal promoters and transgene cassettes. LVs are flanked by the 5′ and 3′ long terminal repeats (LTRs). The EFS-ADA provirus contains the mutated woodchuck hepatitis virus post-transcriptional regulatory element (wPRE) and codon-optimized ADA gene cassette driven by the EFS promoter. The Lenti/βAS3-FB provirus contains the human β-globin promoter (P) and the locus control region (LCR) hypersensitive sites (HSs), HS2, HS3, and HS4 in anti-sense orientation driving the expression of the β-globin gene cassette with 3′ untranslated region (UTR) enhancer. The 3′ LTR of Lenti/βAS3-FB contains the 77-bp FB (FII-BEAD) insulator.

(B) Viral titers of unconcentrated LVs (bars represent mean with SEM; n = 6–9 from three independent experiments; Wilcoxon rank-sum test; $p < 0.001$). LVs in viral supernatant were assayed for titer by transducing HT29 cells at 10-fold serial dilution and vector copy number (VCN) measured by ddPCR. The MND-GFP (3.4 kb) provirus contains the GFP gene cassette driven by a ubiquitous, gamma-retroviral promoter, MND. Empty particle (EPs) are vectors packaged without vector transfer plasmids.

(C) Transduction of human bone marrow (BM) CD34+ HSPCs by LVs at three doses of transduction units (TU) per mL (bars represent mean with SEM; n = 3 independent donors from three independent experiments; linear regression, comparison of slopes, $p < 0.01$). Human BM CD34+ HSPC cells (1×10^6 cells/mL) were prestimulated with cytokines for 24 h and transduced with LVs at three doses of TUs at MOI = 1, 10, and 100 for an additional 24 h. Cells were cultured in an *in vitro* myeloid differentiation condition, and VCN was measured 12 days after transduction.

to quantify initial RNA, PBS primers and probe were used to quantify intermediate RNA, and U3/R primers and probes were used to quantify complete RNA (Figure 2A). RNA was extracted from both packaging cells and vector particles 3 days after transfection in the packaging process. Equal masses of viral RNA were treated with DNase and reverse transcribed with random primers. Amplifications of R/U5, PBS, and U3/R regions were conducted to quantify the initial, intermediate, and complete viral RNA by ddPCR.

As shown in Figure 2B, two simple vectors, EFS-ADA and MND-GFP, displayed similar levels of all three RNA species. The complex vector, Lenti/βAS3-FB, had similar levels of initial and Intermediate RNA as EFS-ADA. Lenti/βAS3-FB had 15.4-fold fewer complete RNA species than EFS-ADA. Only ~9.2% of Lenti/βAS3-FB RNA transcripts initiated at the 5′ LTR has intact 3′ LTR RNA detected by the U3/R primers and probe, whereas ~72.9% viral RNA was complete in EFS-ADA (Figure 2C). The amplification efficiencies by the three sets of primers and probes were assessed using plasmids and gave similar efficiency, suggesting that the decrease in complete RNA in Lenti/βAS3-FB was not a PCR artifact (Figures S2A and S2B).

We further quantified the viral RNA content in vector particles to assess whether the RNA truncation was also observed in vector particles. EFS-ADA and MND-GFP displayed minor decreases in the RNA content from 5′ to 3′, whereas Lenti/βAS3-FB showed a substantial decrease in the level of complete RNA, resulting in 42.9-fold lower complete RNA in Lenti/βAS3-FB than in EFS-ADA (Figure 2D). Lenti/βAS3-FB consisted of only 8% complete viral RNAs in contrast to the 35.9% complete RNA in EFS-ADA, demonstrating that the RNA truncation was observed in both packaging cells and vector particles (Figure 2E).

The R/U5 primer and probes for quantification of initial RNA may additionally pick up readthrough transcripts through the 3′ LTR and introduce a systematic enrichment of R/U5 sequences. To investigate the extent of transcription readthrough, we quantified the abundance of the sequences from the 3′ LTR U5 region to the SV40 polyadenylation site of the plasmid backbone by high-throughput RNA sequencing of the viral RNA (Figure S2D). The readthrough to U5 was relatively low compared with transcription through the 5′ LTR and therefore should not significantly over-estimate the R/U5 reads from the 5′LTR. In addition, the readthrough transcription would be a consistent systematic bias that should not affect the overall conclusions.

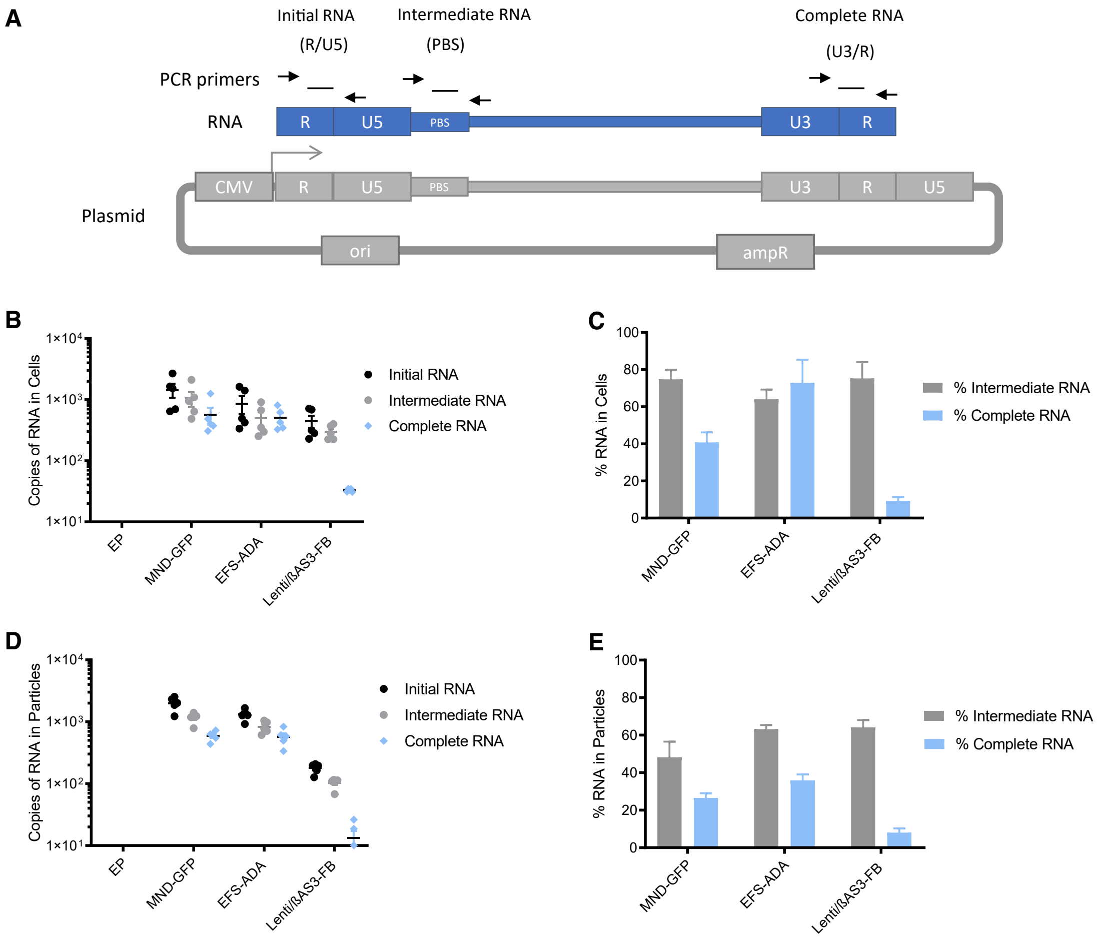

Figure 2. Lenti/βAS3-FB Had Reduced Levels of Complete RNA in Packaging Cells and Vector Particles

(A) Schematic representation of viral RNA and the PCR primers and probes used to quantify each RNA species by ddPCR. Transcription starts at the 5′ LTR driven by the CMV promoter, and RNA is transcribed in the following order: R/U5, PBS, and U3/R. R/U5 primers were used to quantify initial RNA, PBS primers quantify intermediate RNA, and U/3R primers quantify complete RNA.

(B and C) (B) The absolute quantification of viral RNA in 293T packaging cells measured by ddPCR and (C) the percentages of intermediate and complete viral RNA in 293T cells. Cells were harvested 3 days after transfection. Total RNA was extracted from 293T cells, treated with DNase, and reverse transcribed with random primers. The amounts of viral RNA species were quantified by ddPCR. The percentage of intermediate RNA was calculated as intermediate RNA/initial RNA × 100%, and the percentage of complete RNA was calculated as complete RNA/initial RNA × 100%.

(D) The absolute quantification of viral RNA in unconcentrated viral supernatant measured by ddPCR.

(E) The percentages of intermediate and complete RNA in vector particles (B–E, bars represent mean with SEM; n = 5 dishes of identical cultures treated and analyzed in two independent experiments).

RNA Sequencing to Examine the Frequency of the Viral RNA Sequences across the Entire Transcript

We next explored the relationship between vector proviral length and viral RNA transcript completeness. A series of β-globin LVs of different lengths were packaged and titered concurrently, and the levels of complete RNA in the unconcentrated viral supernatant were quantified. As expected, the titer of the LVs negatively correlated with the vector length (Pearson correlation, $r^2 = 0.81$, $p < 0.01$) (Figure 3A). The concentration of complete RNA also decreased with increasing vector length in all LVs (Pearson correlation, $r^2 = 0.68$, $p < 0.05$) (Figure 3B).

The decrease in complete RNA in complex LVs could be caused by the presence of specific sequences, which lead to discrete termination of vector transcripts, such as cryptic polyadenylation signals or splice signals. Alternatively, there may be progressive termination of transcription in a length-dependent manner, consistent with a previous study that demonstrated that inefficient transcription of the HIV provirus is associated with premature termination

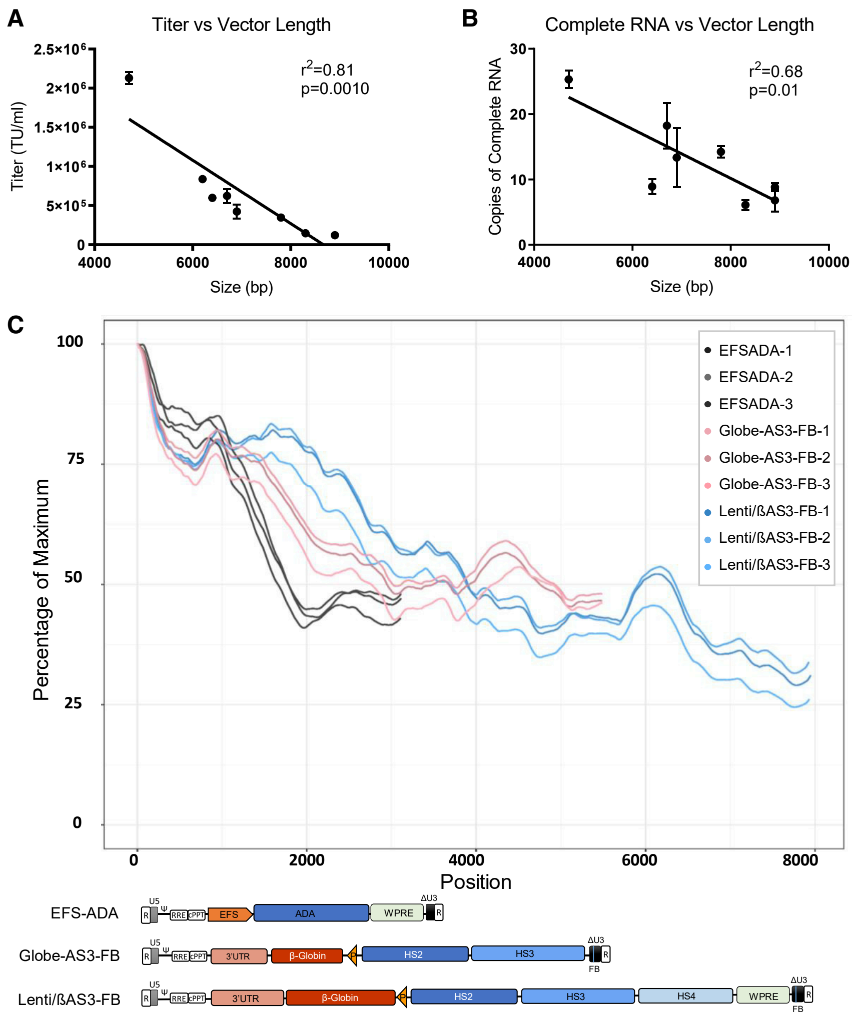

Figure 3. Viral RNA of LVs Showed Length-Dependent Decrease in Completeness and Coverage in RNA Sequencing

(A) Comparison of titer versus vector length (bars represent mean with SEM; n = 3 independent experiments; Pearson correlation, r = −0.86, $p < 0.01$).

(B) Complete RNA versus vector length in a panel of β-globin vectors of different lengths (bars represent mean with SEM; n = 3 independent experiments; Pearson correlation exclude Globe-AS3, r = −0.67, $p < 0.05$). All β-globin vectors were packaged and assayed concurrently. Viral RNA was extracted from vector particles.

(C) Percentage of maximum reads of EFS-ADA, Globe-AS3-FB, and Lenti/βAS3-FB versus vector length (n = 3 independent experiments). Schematic representation of the viral RNA is displayed below. LVs were packaged in three independent experiments. Total RNA was extracted from 140 μL unconcentrated viral supernatant followed by DNase treatment. RNA was fragmented to an average of 400 bp, reverse transcribed by random priming, and sequencing adaptors ligated. The library was sequenced on an Illumina HiSeq 3000.

of viral RNA, attributed to poor processivity of RNA polymerase II (RNAP II) (Zhang et al., 2007). To identify the mechanisms that truncate viral RNA, we characterized the frequency of the viral RNA sequences across the entire transcripts using Illumina RNA sequencing.

Viral RNA was extracted from unconcentrated viral supernatants packaged from three independent experiments, and sequencing library preparations were produced and sequenced by the UCLA Technology Center for Genomics and Bioinformatics. Two β-globin LVs of different lengths, Globe-AS3-FB (6.4 kb) and Lenti/βAS3-FB (8.9 kb), and the EFS-ADA LV (4.2 kb) were analyzed. Reads were aligned to the vector genome and plotted as the percentage of maximum reads versus mapping position of the vector genome (Figure 3C). All LVs had the most abundant reads at the 5′ LTR, which progressively decreased in abundance toward the 3′ end. No discrete termination of viral transcripts was observed, suggesting that the termination was not caused by the presence of specific problematic sequences. The reads of both Globe-AS3-FB and EFS-ADA maintained at ~50% of the maximum reads at the 3′ LTR, whereas Lenti/βAS3-FB experienced a further decrease after 6 kb, resulting in ~25–30% RNA with 3′ LTR. The decrease in coverage from 6 to 9 kb in the two β-globin vectors with similar sequences but different length suggested that the RNA truncation is length dependent and is likely to be caused by poor processivity of RNAP II.

Truncated RNA Failed Reverse Transcription at the First-Strand Transfer

We next investigated the reverse transcription kinetics of Lenti/βAS3-FB and EFS-ADA in target cells to elucidate the inhibitory effect of truncated RNA. The steps of reverse transcription, locations of ddPCR primers and probes, and the reverse-transcribed products are illustrated in Figure S3A. A tRNA serves as a primer and binds to a

complementary sequence on the 5′ end of the viral RNA genome termed as the PBS to initiate reverse transcription (first-strand priming). First-strand synthesis proceeds from 5′ to 3′ to make the single-stranded DNA copy of the R and U5 regions, and then RNAse H degrades the R and U5 regions on the 5′ end of the viral RNA. The newly formed first-strand viral DNA then finds the complementary R on the 3′ end of the viral genome (first-strand transfer) and resumes the first-strand synthesis from 5′ to 3′ using the viral RNA as a template. First-strand viral DNA elongates to generate the U3 region first and, at the end, the Psi region. Therefore, primer and probes spanning the R/U5 region were designed to quantify early reverse-transcribed products (first-strand priming), primer and probes spanning the U3/R region were designed to quantify intermediate reverse-transcribed products (first-strand transfer), and primer and probes spanning the Psi region were designed to quantify late reverse-transcribed products (first-strand elongation). Since the R sequence in the 3′ LTR is absent in truncated RNA, we hypothesized that incomplete RNA in Lenti/βAS3-FB failed reverse transcription at the first-strand transfer step (Figure S3B).

A primitive human hematopoietic myeloid progenitor cell line, KG1a, was chosen as the target cells because of its relative resistance to LV transduction, similar to that of primary human CD34+ HSPCs (Koeffler et al., 1980). A control vector, bovine growth hormone polyadenylation sequence (BGH-ADA), was a truncated version of EFS-ADA made by inserting the BGH-ADA signal upstream of the 3′ LTR (Figure S3C). KG1a cells were transduced with equal amounts of initial viral RNA of Lenti/βAS3-FB, EFS-ADA, and BGH-ADA vector particles and collected 24 h after infection for analysis of serial reverse transcription products.

As expected, all three vectors displayed similar kinetics for the production of early reverse-transcribed products, because equal amounts of initial RNA were used to infect cells (Figure S3D). Interestingly, compared with EFS-ADA, Lenti/βAS3-FB produced 4-fold fewer intermediate reverse-transcribed products and 3-fold fewer late reverse-transcribed products, similar to the truncated vector BGH-ADA. These data strongly suggest that truncated RNA failed to find the complementary R at the 3′ LTR of the viral RNA and resulted in premature termination of reverse transcription at the first-strand transfer step. BGH-ADA displayed low levels of intermediate and late reverse-transcribed products. It was likely to be caused by the transcriptional readthrough of the polyadenylation signal, and full-length RNA was produced at a low level.

Shortening the Vector Length Increased Viral RNA, Titer, and Gene Transfer

We next sought methods to improve titer and infectivity of the complex β-globin vector by rescuing the viral RNA truncation. Because the level of complete RNA decreases with increasing vector length, we hypothesized that shortening the vector can rescue RNA production, thereby increasing titer and infectivity. Morgan et al. (2019) reported a 5.3-kb β-globin vector "Mini-G," where the major size reductions are caused by using short LCR enhancer regions identified from the ENCODE database. To test our hypothesis, we packaged Mini-G and Lenti/βAS3-FB concurrently and measured the RNA content in viral particles, titer, and infectivity in CD34+ BM cells (Figure 4A).

Shortening the vector successfully rescued the RNA production and increased titer and infectivity in CD34+. Mini-G showed 27-fold more complete RNA than Lenti/βAS3-FB (unpaired t test, $p = 0.0003$) (Figure 4B). The percentage of complete RNA increased from 5.4% in Lenti/βAS3-FB to 84% in Mini-G, which led to a 15.5-fold increase in the percentage of complete RNA (unpaired t test, $p < 0.0001$). In addition, viral titer of the unconcentrated virus was 7.6-fold higher for Mini-G (unpaired t test, $p = 0.0004$) (Figure 4C). Mini-G also showed an improvement in the infectivity of human BM CD34+ HSPCs (linear regression, comparison of the slopes, $p = 0.0007$) (Figure 4D).

Packaging with Tat Increased Viral RNA

Tat is an accessory protein encoded in the genomes of wild-type HIV-1 that plays a pivotal role in transcription activation and elongation. Upon binding to the Tat-responsive element (TAR) hairpin from the 5′ end of the HIV transcript, Tat recruits the transcriptional elongation factor pTEFb to phosphorylate the C-terminal domain of RNAP II, thereby enhancing the processivity of the polymerase (Parada and Roeder, 1996; Kao et al., 1987; Romani et al., 2010; Feinberg et al., 1991; Laspia et al., 1989). We hypothesized that packaging with Tat will increase the production of complete RNA in LV. To test our hypothesis, we packaged Lenti/βAS3-FB, EFS-ADA, and DL-βAS3 vectors with addition of either a Tat expression plasmid or a filler plasmid (unpackageable GFP plasmid without lentiviral genome). The Lenti/βAS3-FB vector plasmid is driven by the CMV promoter, whereas DL-βAS3, the parent LV to Lenti/βAS3-FB, contains an intact 5′ HIV-1 LTR and is thus Tat dependent.

We first assessed the effect of Tat on viral RNA production. Packaging with the addition of the Tat plasmid increased the levels of all viral RNA species from all three vectors (Figure 5A). In particular, we observed a 2- to ~3-fold increase in the absolute quantity of complete RNA from EFS-ADA and Lenti/βAS3-FB and a ~6-fold increase in complete RNA from DL-βAS3 (unpaired t test, *$p < 0.05$, **$p < 0.01$, ***$p < 0.001$). Tat significantly increased the percentage of complete RNA in DL- βAS3 (unpaired t test, $p = 0.02$), confirming that Tat was necessary for efficient transcription elongation from LV with intact 5′ HIV

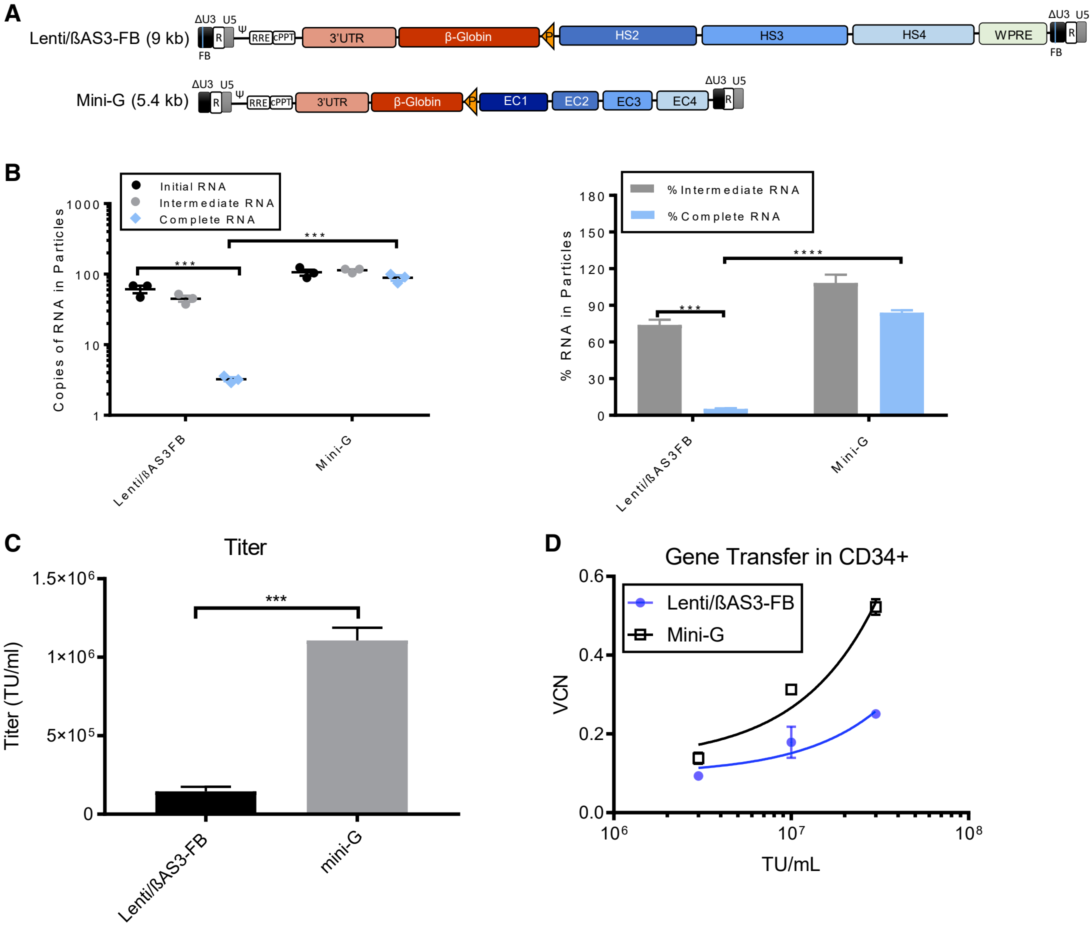

Figure 4. Shortening the Vector Length Increased Viral RNA Completeness, Vector Titer, and CD34+ Cell Infectivity

(A) The schematic representation of the provirus of Lenti/βAS3-FB and Mini-G.

(B) The absolute quantification and percentage of viral RNA in viral particles measured by ddPCR.

(C) Viral titers of Lenti/βAS3-FB and Mini-G (B and C, bars represent mean with SEM; n = 3 independent experiments; unpaired t test, *$p < 0.05$, **$p < 0.01$, ***$p < 0.001$, ****$p < 0.0001$).

(D) Infectivity of Lenti/βAS3-FB and Mini-G LVs in 1×10^6 cells/mL of BM CD34+ HSPCs at MOI = 3, 10, and 30 (bars represent mean with SEM; n = 3 independent experiments; linear regression, comparison of the slopes, p = 0.0007). Data represent measurements from three independent human BM CD34+ HSPC donors.

LTR in the packaging plasmid (Figure 5B). However, Tat did not increase the percentage of complete RNA from EFS-ADA or Lenti/βAS3-FB, suggesting that the CMV-driven LV packaging plasmids are not Tat dependent for transcription elongation.

Nevertheless, we examined whether the increase in the absolute quantities of full-length viral RNA when packaged with Tat correlate with viral titer. The titers of EFS-ADA and Lenti/βAS3-FB increased by 2- to ~3-fold, and the titer of DL-118 increased by ~6-fold (unpaired t test, EFS-ADA p = 0.01, DL- βAS3 p = 0.004, Lenti/βAS3-FB p = 0.39) (Figure 5C). Although the increase in titer for Lenti/βAS3-FB was not statistically significant, we observed a consistent 2-fold increase in titer in LVs packaged with Tat in each independent experiment. Furthermore, LVs packaged with Tat, especially DL- βAS3 and EFS-ADA, showed an improvement in their infectivity in human BM CD34+ HSPCs (linear regression, comparison of the slopes, EFS-ADA p = 0.039, DL- βAS3 p = 0.01, Lenti/βAS3-FB p = 0.105) (Figure 5D).

We tried to further increase the level of complete RNA by manipulating the location of the Rev-response element (RRE). The HIV-1 Rev protein binds to the RRE to facilitate nuclear export of viral RNA (Cullen, 2005; Mann et al., 1994; Battiste et al., 1996; Pollard and Malim, 1998). Moving RRE from downstream of the 5′ LTR to a position upstream of the 3′ LTR modestly increased the copies of complete RNA without changing the percentage of complete RNA and had no significant effect on titer (Figure S4).

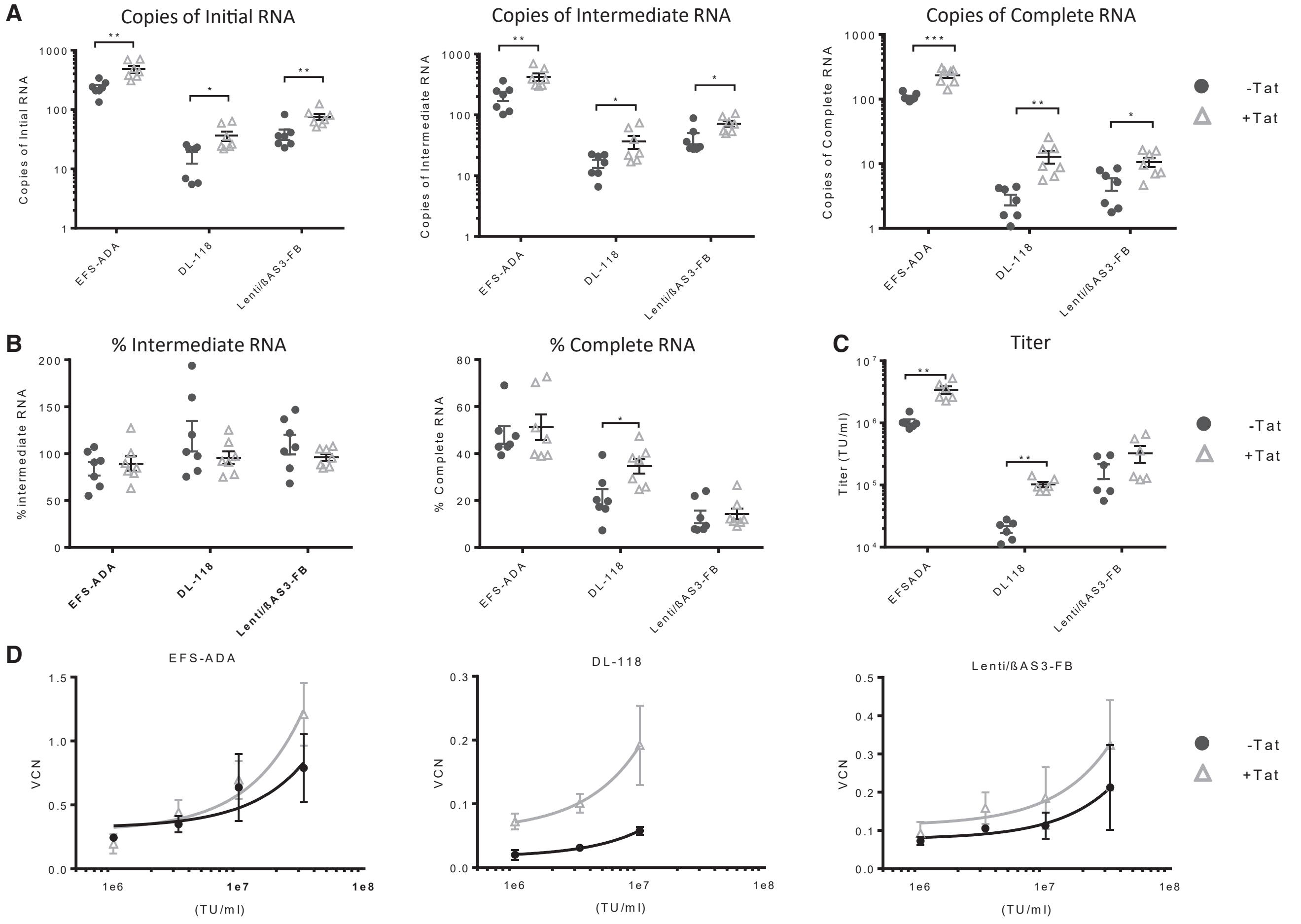

Figure 5. Packaging with Tat Increased Viral RNA Amounts but Not Completeness, Vector Titer, and CD34+ Cell Infectivity

(A and B) (A) The absolute quantification and (B) percentage of initial, intermediate, and complete RNA in EFS-ADA, DL- βAS3, Lenti/βAS3-FB LVs. DL-βAS3 (a parental β-globin vector to Lenti/βAS3-FB) contains an intact 5′ HIV LTR and is dependent on Tat for full-length RNA transcription. Vectors were packaged with either Tat plasmids or a filler plasmid (an unpackageable GFP plasmid without lentiviral sequences) and harvested 3 days after transfection. Viral RNA was extracted from vector particles.

(C) Titers of LVs packaged +/− Tat plasmids (A–C, bars represent mean with SEM; n = 6 dishes of identical cultures from three independent experiments; unpaired t test, $^{*}p < 0.05$, $^{**}p < 0.01$, $^{***}p < 0.001$).

(D) Infectivity of CCLAS3 and Globe LVs +/− Tat in 1×10^6 cells/mL of BM CD34+ HSPCs at MOI = 1, 3, 10, and 30 (bars represent mean with SEM; n = 3 independent experiments; linear regression, comparison of the slopes, EFS-ADA p = 0.039, DL-βAS3 p = 0.01, Lenti/βAS3-FB p = 0.105). Data represent measurements from three independent human BM CD34+ HSPC donors.

Knocking out *PKR* in Packaging Cells Rescued Vector Protein Production and Increased Titer and Infectivity of Reverse Orientation Vectors

Although EFS-ADA and Lenti/βAS3-FB had similar levels of initial and Intermediate RNA in 293T packaging cells, Lenti/βAS3-FB had substantially reduced initial and intermediate RNA species in viral supernatant compared with EFS-ADA, as shown in Figures 2B and 2D. We therefore assessed the RNA export efficiency from cells to virions as indicated by the ratios of copies of initial RNA in vector particles to copies of initial RNA in packaging cells (Figure 6A). Surprisingly, the export efficiency was 4.6-fold higher for EFS-ADA than for Lenti/βAS3-FB. We next assessed the physical vector particle production by measuring the p24 concentration in viral supernatant. Surprisingly, despite the fact that equal amounts of Gag/Pol plasmids were used at packaging, the p24 concentration of Lenti/βAS3-FB was considerably lower than the p24 concentrations of the three controls, including empty particles, which were packaged without vector transfer plasmids (Figure 6B). When we normalized viral RNA export efficiency to p24 concentration, EFS-ADA and Lenti/βAS3-FB had nearly

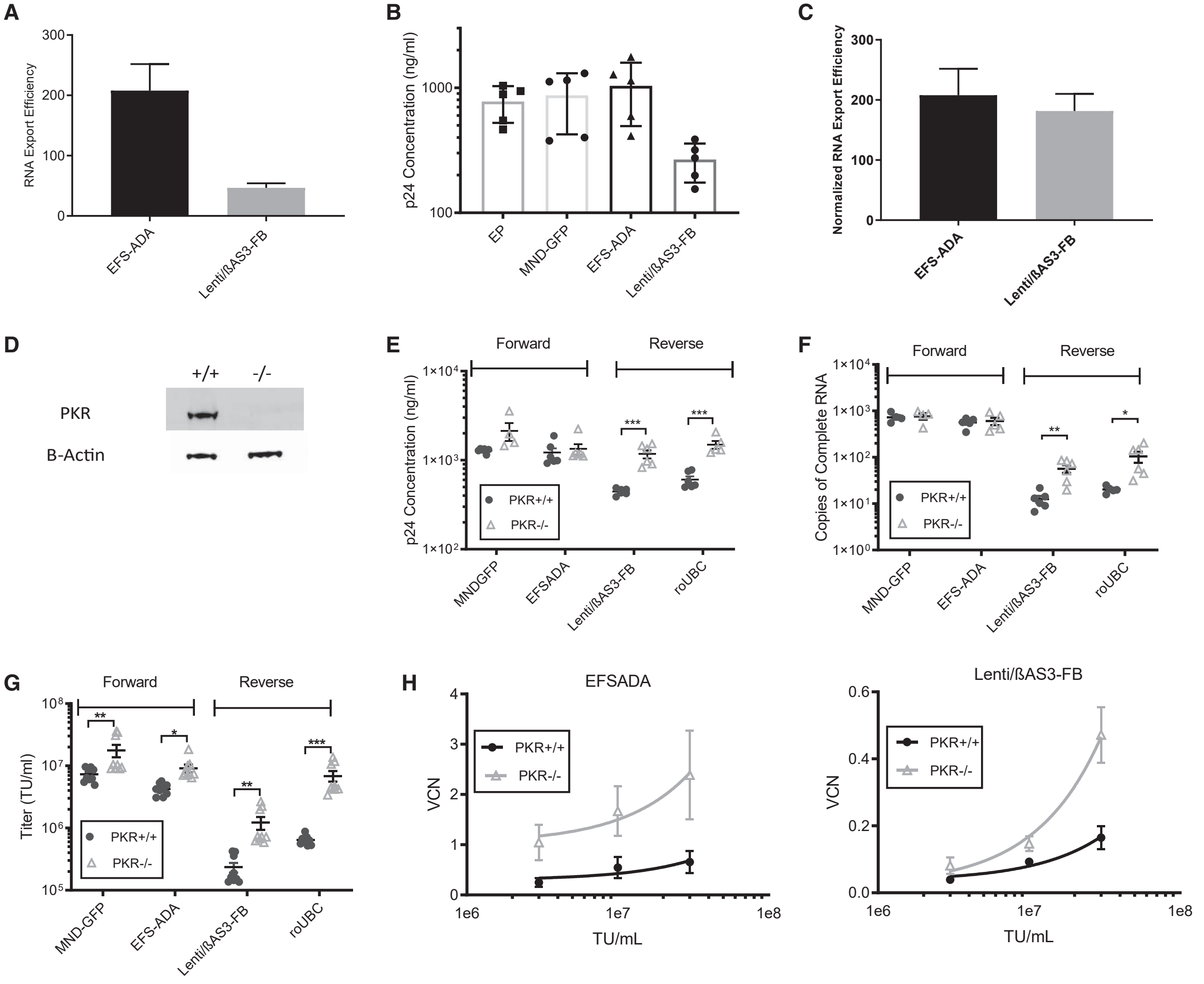

Figure 6. Knocking out *PKR* in 293T Cells Rescued p24 Production and Increased Vector Titers and CD34+ Cell Infectivity

(A) The export efficiency of initial RNA from cells to vector particles of EFS-ADA and Lenti/βAS3-FB. RNA export efficiency was defined as the ratios of copies of initial RNA in vector particles to copies of initial RNA in packaging cells.

(B) p24 concentration quantified by ELISA of EP (empty particle), MND-GFP, EFS-ADA, and Lenti/βAS3-FB unconcentrated viral supernatant.

(C) The export efficiency normalized for the fold difference in p24 concentration (A–C, bars represent mean with SEM; n = 5 dishes of identical cultures from two independent experiments).

(D–G) (D) PKR protein expression in parental and PKR−/− cells measured by western blot. PKR was knocked out by CRISPR-Cas 9, and a single-cell clone with no parental PKR alleles was expanded for protein expression analysis by western blot. β-Actin was used as the loading control. LVs were packaged in either parental or PKR−/− (KO) 293T cells. Viral supernatant was harvested for (E) p24 concentration, (F) the absolute quantification of complete RNA, and (G) viral titers (E–G, bars represent mean with SEM; unpaired t test, *$p < 0.05$, **$p < 0.01$, ***$p < 0.001$; n = 4–9 dishes of identical cultures from three independent experiments).

(H) Infectivity of EFS-ADA and Lenti/βAS3-FB packaged either in parental or PKR−/− 293T cells in three independent human BM CD34+ HSPCs (bars represent mean with SEM; n = 3 independent experiments; linear regression, comparison of the slopes, EFS-ADA p = 0.29, Lenti/βAS3-FB p = 0.0016). HSPCs (1×10^6 cells/mL) were transduced at MOI = 3, 10, 30.

equal RNA export efficiency from cells to particles, suggesting that RNA export was not the primary limiting factor (Figure 6C). Instead, physical particle formation by Lenti/βAS3-FB seemed to be impaired.

It is conceivable that Lenti/βAS3-FB, but not EFS-ADA and MND-GFP, triggered a cellular response to inhibit p24 production. PKR is an interferon-induced, double-stranded RNA (dsRNA)-activated protein kinase that protects cells against viral infections (Pindel and Sadler, 2011). Vectors with an internal expression cassette in the reverse orientation were shown by Kafri and colleagues to trigger PKR response to inhibit protein translation (Hu et al., 2018; Poling et al., 2017). To test whether PKR was activated by Lenti/βAS3-FB, we knocked out *PKR* in 293T cells via CRISPR-Cas9, generated an isogenic *PKR*−/− cell clone, and confirmed the allelic disruption by TIDE sequencing and protein expression by western blot (Figures 6D, S5A, and S5B).

Two forward-oriented vectors bearing expression cassettes in the sense direction, MND-GFP and EFS-ADA, and two reverse-oriented vectors bearing expression cassettes in the anti-sense direction, Lenti/βAS3-FB and roUBC, were packaged in either parental 293T cells or *PKR*−/− 293T cells. roUBC (4.2 kb) consists of a reverse-oriented GFP expression cassette driven by the Ubiquitin C (UBC) promoter.

Knocking out *PKR* restored the p24 production by both reverse-oriented vectors (unpaired t test, ***$p < 0.001$) (Figure 6E). In addition, packaging in *PKR*−/− cells significantly increased the level of all three RNA species from either reverse-oriented vectors without changing the percentage of RNA (unpaired t test, *$p < 0.05$, **$p < 0.01$, ***$p < 0.001$) (Figures 6D, S5C, and S5D). Packaging in *PKR*−/− cells led to a 5-fold increase in titer for Lenti/βAS3-FB and an 11-fold increase in titer for roUBC, and the titer of the reverse-oriented GFP vector roUBC matched to the titer of the forward-oriented GFP vector MND-GFP (unpaired t test, *$p < 0.05$, **$p < 0.01$, ***$p < 0.001$) (Figure 6G). Notably, packaging in *PKR*−/− cells also increased the titer of the forward-oriented vectors by 2- to 3-fold (unpaired t test, MND-GFP $p = 0.002$, EFS-ADA $p = 0.02$). These data strongly suggest that PKR was activated and inhibited the p24 production in reverse-oriented vectors. The increase in titer of forward-oriented vectors was likely caused by overcoming the antiviral effect of PKR recognizing single-stranded viral RNA species from these vectors, such as HIV TAR (Samuel, 2001).

Finally, we characterized the infectivity of the LVs packaged in *PKR*−/− cells in human BM CD34+ cells. We observed an increase in infectivity of both EFS-ADA and Lenti/βAS3-FB LVs that were packaged in *PKR*−/− cells compared with the LVs packaged in parental 293T cells (linear regression, comparison of the slopes, EFS-ADA $p = 0.29$, Lenti/βAS3-FB $p = 0.0016$) (Figure 6H).

By combining all three modifications reported in this paper (shortening the vector length; packaging with Tat; and packaging in *PKR*−/− cells), we were able to increase the copies of complete RNA from the Mini-G vector compared with Lenti/βAS3-FB packaged without Tat in PKR-replete cells by 149.9-fold and to improve the percentage of Complete RNA to 79.4%. These strategies resulted in a 29.3-fold increase in titer (Figure S6) and increased the infectivity of the β-globin vector in human BM CD34+ HSPCs by 3.2-fold at the highest dose (bars represent mean with SEM; n = 3 independent experiments; linear regression, comparison of slopes, $p = 0.0009$).

DISCUSSION

The advances in clinical and scientific understanding of lentiviral gene therapy in the past decades have enabled the first wave of clinical gene therapy successes for multiple diseases (Aiuti et al., 2009; Biffi et al., 2011; Cartier et al., 2012; De Ravin et al., 2016). However, low titer and poor gene transfer of complex vectors remain critical challenges for successful clinical application of these vectors. Viral titer is a critical factor in the costs of production, and the titers of complex vectors are typically 1–2 log-orders lower than the titers of simple vectors. In addition, even when adjusted to the same TU, transduction of HSPCs with complex vectors results in lower VCNs that barely increase with the use of more infectious particles. The low titer and poor gene transfer may be caused by the lengthy and complex nature of the genome, but the exact mechanisms that restrict complex vectors to perform as efficiently as simple GFP vectors have remained elusive. Here, we demonstrated that viral RNA and protein production were impaired in complex vectors, such as Lenti/βAS3-FB.

The majority of viral RNA of Lenti/βAS3-FB was truncated and was not fully reverse transcribed, thus resulting in fewer complete linear forms of viral DNA for nuclear translocation and integration. The RNA truncation was observed in a length-dependent manner, suggesting that the truncation may be caused by the poor processivity of RNAP II during transcription elongation. In fact, multiple studies demonstrated that inefficient transcription of the HIV provirus is associated with premature termination of transcription (Zhang et al., 2007; Kao et al., 1987; Feinberg et al., 1991; Laspia et al., 1989; Cullen, 1990).

We used two strategies to increase viral RNA production: shortening the vector length to ensure that viral RNA can be fully transcribed and packaging with HIV Tat to enhance the processivity of RNAP II. Shortening the 8.9-kb Lenti/βAS3-FB vector to the 5.3-kb Mini-G vector rescued the production of complete RNA, resulting in a 7.6-fold increase in titer and 2-fold increase in CD34+ cell infectivity at the

highest dose. Packaging LVs with Tat improved RNA initiation but not RNAP II processivity from CMV-driven lentiviral vector packaging plasmids. Nonetheless, a 2-fold increase in titer and infectivity at the high dosage of TU was observed in Lenti/βAS3-FB when packaged with Tat. Future studies to improve RNAP II processivity or to identify additional problematic sequences that truncate RNA are needed.

We also observed that Lenti/βAS3-FB triggered an antiviral response mediated by PKR to inhibit virion formation. dsRNA-activated PKR inactivates the eukaryotic translation initiation factor 2A, resulting in the inhibition of mRNA translation initiation (Pindel and Sadler, 2011; Samuel, 2001; Garcia et al., 2006). Although LVs are single-stranded RNA viruses, non-lineage-specific activation of the internal β-globin promoter in the reverse-oriented Lenti/βAS3-FB may lead to the production of viral RNA in the anti-sense orientation; hybridization of the sense RNA driven by the CMV promoter and anti-sense RNA driven by the internal promoter can lead to the production of dsRNA and PKR response. Our observations were consistent with others that bidirectional vectors had low titer due to the formation of dsRNA (Maetzig et al., 2010).

Previous studies have showed that knocking out certain cellular factors improved the production of LVs (Hu et al., 2018; Park et al., 2018). In this paper, we showed that knocking out PKR in the packaging cells restored p24 production in the reverse-oriented vectors, suggesting that PKR was activated to inhibit P24 production in reverse-oriented LVs. Furthermore, we demonstrated potentially additive effects of combining the three strategies: shortening the vector, packaging with Tat, and knocking out PKR to increase titer by ~30-fold and infectivity by 3-fold for the β-globin vector. These modifications were able to increase the production of viral substrates that are essential for the remaining steps of lentiviral life cycle.

In summary, the complex and lengthy nature of the viral genome of Lenti/βAS3-FB led to RNA truncation in a length-dependent manner and triggered interferon response to inhibit viral protein production during the packaging phase. We reported three strategies in this study—shortening the vector length, packaging with Tat, and packaging in *PKR−/−* cells—to overcome current limitations with the complex LV and these may further support clinical translation of novel lentiviral autologous gene therapy for treating genetic blood cell disorders.

EXPERIMENTAL PROCEDURES

LV Production and Titration

LVs used in this study included EFS-ADA, Lenti/βAS3-FB, Globe-AS3, DL- βAS3, Lenti/βAS3-FB (-HS4/+HPRT), Lenti/βAS3-FB (-HS4), CoreGA-AS3-FB, Core-AS3-FB, UV-AS3, Mini-G, roUBC, MND-GFP, and a version of EFS-ADA designed to not generate any full-length LV genomes by inserting the BGH-ADA upstream of the 3′ LTR (Figure S1) (Carbonaro et al., 2014; Cooper et al., 2015; Levasseur et al., 2003; Morgan et al., 2019; Romero et al., 2013; Urbinati et al., 2018).

LVs were packaged by transient transfection of 293T cells with fixed amounts of HIV Gag/Pol, Rev, and VSV-G expression plasmids and equimolar amounts of each of the different vector transfer plasmids using TransIT-293 (Mirus Bio, Madison, WI) as described in the Supplemental Methods and Cooper et al. (2011). For some experiments, cells were co-transfected with addition of either the HIV-1 pSV2-TAT expression plasmid (NIH AIDS Reagent Program, Rockville, MD) or, as a control, a GFP expression plasmid lacking vector elements so that it would not be packaged.

To determine the titers of the LVs, the HT-29 human colon carcinoma cell line was transduced with different dilutions of the LVs and harvested ~60 h after transduction. Genomic DNA was extracted using the PureLink Genomic DNA Mini Kit (Invitrogen, Waltham, MA). VCN was measured by droplet digital PCR (ddPCR), as described previously in Masiuk et al. (2019), and viral titers were calculated based on VCN.

LV Infection in Human BM CD34+ HSPCs

All human samples have been used following UCLA IBC protocol 2019-126-001. Human CD34+ HSPCs were isolated from healthy donor BM aspirates using Ficoll-Hypaque gradient separation and a CD34 MicroBead Kit (Miltenyi Biotech, Bergisch Gladbach, Germany). The processed cells were cryopreserved in liquid nitrogen with BAMBANKER (Wako Chemicals, VA, USA).

CD34+ HSPCs were thawed at 37°C and plated at 1×10^6 cells/mL in non-tissue culture-treated plates coated with RetroNectin (20 μg/mL; Takara Shuzo, Otsu, Japan). The cells were prestimulated for 24 h in X-Vivo 15 (Lonza, Basel, Switzerland) with 1× L-glutamine-penicillin-streptomycin (Gemini Bio-Products, West Sacramento, CA, USA), 50 ng/mL stem cell factor (SCF), 50 ng/mL thrombopoietin (TPO), 50 ng/mL Flt3L, and 20 ng/mL IL-3 (PeproTech, Rocky Hill, NJ, USA). Prestimulated cells were transduced with concentrated viral supernatants, and 24 h after transduction cells were collected for *in vitro* myeloid differentiation cultures, as described below.

In Vitro Myeloid Differentiation Cultures

Transduced human BM CD34+ HSPCs were cultured in basal BM medium ([BBMM]: Iscove's modified Dulbecco's medium [IMDM] [Life Technologies, Grand Island, NY], 1× L-glutamine-penicillin-streptomycin, 20% fetal bovine serum, 0.52% BSA) with recombinant human cytokines of 5 ng/mL interleukin-3, 10 ng/mL interleukin-6, and 25 ng/mL cKit ligand (hSCF) (PeproTech) at 37°C, 5% CO_2. Cells were maintained in culture for 12 days with addition of fresh BBMM and cytokines every 4 days. After 12 days, the cells were harvested for genomic DNA extraction and VCN analysis by ddPCR as described above.

Viral RNA Analysis by ddPCR

RNA was extracted from either 2×10^6 293T cells or 140 μL unconcentrated viral supernatant 3 days after transfection for vector packaging. Cellular RNA was extracted using the RNeasy Plus Mini Kit (QIAGEN, Hilden, Germany), and RNA from the viral

supernatant was extracted using the QIAmp Viral RNA Mini Kit (QIAGEN). Equal masses of RNA were treated with DNase I (Invitrogen) to remove any traces of genomic or plasmid DNA contamination and then reverse transcribed using random primers, M-MLV reverse transcriptase, and RNAseOUT Recombinant Ribonuclease Inhibitor (all from Invitrogen), following the manufacturer's protocol. Amplification of R/U5, PBS, and U3/R regions by ddPCR was conducted to quantify the initial, intermediate, and complete viral RNA. The cycling conditions were 95°C for 10 min for one cycle (94°C for 30 s and 60°C for 1 min) for 40 cycles, 10 min at 98°C for one cycle, and a 12°C hold.

p24 Assay

p24 antigen concentration in vector supernatants were measured by the UCLA/CFAR (Center for AIDS Research) Virology Core using the Alliance HIV-1 p24 Antigen ELISA Kit (cat. no. NEK050, PerkinElmer, Waltham, MA), following the manufacturer's manual.

RNA Sequencing

Total RNA was extracted from raw viral supernatant using the QIAmp Viral RNA Mini Kit (QIAGEN). Viral RNA was treated with Turbo DNase (Invitrogen) and sent to the UCLA Technology Center for Genomics and Bioinformatics Core for downstream library preparation. A cDNA library was prepared using the KAPA RNA HyperPrep Kit with RiboErase (Roche Sequencing, Pleasanton, CA) following the manufacturer's manual without rRNA depletion to avoid bias. In brief, RNA was fragmented into ~400-bp fragments; first-strand cDNA was synthesized with random priming followed by second-strand synthesis. Paired end reads (150 bp) were sequenced on a HiSeq 3000 (Illumina, San Diego, CA). The reads were aligned to the designed vectors via a Burrows-Wheeler Aligner (version 0.7.17) using parameters "bwa mem -t 16 -k 100 -A 1 -B 30 -O 10 -E 10." Bigwig files were generated on the bam files using the bamCoverage function from deepTools. The numbers of reads for each location were extracted from the bigwig files into R, and the average of the upstream and downstream 450 bp were calculated as the coverage score. The coverage scores were then normalized to the maximum value for each sample.

Statistical Analysis

Descriptive statistics, such as number of observations, mean, and standard error are reported and presented graphically for quantitative measurements. Unpaired t tests were used to compare between vectors for outcome measures, such as titers, VCN, copies, and percentage of initial/intermediate/complete RNA. In the case of normality assumption violation, nonparametric Wilcoxon rank-sum tests were used. Evaluation of infectivity of vectors was done by comparing the slopes of respective regression lines. Pearson's correlation was used to correlate the titer of the LVs with the proviral length. For all statistical investigations, tests for significance were two tailed. A p value of less than the 0.05 significance level was considered to be statistically significant. All statistical analyses were carried out using statistical software SAS version 9.4 (SAS Institute, 2013) and GraphPad Prism version 8.3.0 (GraphPad Software, San Diego, CA, USA).

Data and Code Availability

The RNA sequencing data can be accessed via the GEO repository number GEO: GSE158252.

SUPPLEMENTAL INFORMATION

Supplemental Information can be found online at https://doi.org/10.1016/j.stemcr.2020.10.007.

AUTHOR CONTRIBUTIONS

J.H., R.P.H., and D.B.K. conceived and designed all experiments. M.P. and M.M. provided advice on portions of the experiments. J.H. executed and analyzed all experiments. J.H., K.T., C.T., B.A., and J.Q. helped execute portions of the experiments. R.P.H. provided research materials. R.P.H. and D.B.K. designed the experiments. F.M and X.W. performed bioinformatics analysis. D.B.K. provided financial and administrative support. J.H. and D.B.K. wrote the manuscript. J.H. and D.B.K. approved the final manuscript.

ACKNOWLEDGMENTS

This work was supported by NIH T32 Interdisciplinary Training in Virology and Gene Therapy Training grant (T32 AI060567, to J.H.). The Flow Cytometry Core of the UCLA Eli and Edythe Broad Center of Regenerative Medicine and Stem Cell Research, the Virology Core of the UCLA Center for AIDS Research (5P30 AI028697) and the UCLA Technology Center for Genomics & Bioinformatics were used to support studies.

Received: May 6, 2020
Revised: October 15, 2020
Accepted: October 16, 2020
Published: November 12, 2020

REFERENCES

Aiuti, A., Cattaneo, F., Galimberti, S., Benninghoff, U., Cassani, B., Callegaro, L., Scaramuzza, S., Andolfi, G., Mirolo, M., Brigida, I., et al. (2009). Gene therapy for immunodeficiency due to adenosine deaminase deficiency. N. Engl. J. Med. *360*, 447–458.

Battiste, J.L., Mao, H., Rao, N.S., Tan, R., Muhandiram, D.R., Kay, L.E., Frankel, A.D., and Williamson, J.R. (1996). α Helix-RNA major groove recognition in an HIV-1 Rev peptide-RRE RNA complex. Science *273*, 1547–1551.

Biffi, A., Bartholomae, C.C., Cesana, D., Cartier, N., Aubourg, P., Ranzani, M., Cesani, M., Benedicenti, F., Plati, T., Rubagotti, E., et al. (2011). Lentiviral vector common integration sites in preclinical models and a clinical trial reflect a benign integration bias and not oncogenic selection. Blood *117*, 5332–5339.

Carbonaro, D.A., Zhang, L., Jin, X., Montiel-Equihua, C., Geiger, S., Carmo, M., Cooper, A., Fairbanks, L., Kaufman, M.L., Sebire, N.J., Hollis, R.P., et al. (2014). Preclinical demonstration of lentiviral vector-mediated correction of immunological and metabolic abnormalities in models of adenosine deaminase deficiency. Mol. Ther. *22*, 607.

Cartier, N., Hacein-Bey-Abina, S., Bartholomae, C.C., Bougnres, P., Schmidt, M., Von Kalle, C., Fischer, A., Cavazzana-Calvo, M., and Aubourg, P. (2012). Lentiviral hematopoietic cell gene therapy for X-linked adrenoleukodystrophy. In Methods in Enzymology (Academic Press Inc.), pp. 187–198.

Cooper, A.R., Lill, G.R., Gschweng, E.H., and Kohn, D.B. (2015). Rescue of splicing-mediated intron loss maximizes expression in lentiviral vectors containing the human ubiquitin C promoter. Nucleic Acids Res. *43*, 682–690.

Cooper, A.R., Lill, G.R., Gschweng, E.H., and Kohn, D.B. (2011). Highly efficient large-scale lentiviral vector concentration by tandem tangential flow filtration. J. Virol. Methods *177*, 1–9.

Counsell, J.R., Asgarian, Z., Meng, J., Ferrer, V., Vink, C.A., Howe, S.J., Waddington, S.N., Thrasher, A.J., Muntoni, F., Morgan, J.E., et al. (2017). Lentiviral vectors can be used for full-length dystrophin gene therapy. Sci. Rep. *7*, 79.

Cullen, B.R. (2005). Nuclear RNA export unwound. Nature *433*, 26–27.

Cullen, B.R. (1990). The HIV-1 Tat protein: an RNA sequence-specific processivity factor. Cell *63*, 655–657.

De Ravin, S.S., Wu, X., Moir, S., Anaya-O'Brien, S., Kwatemaa, N., Littel, P., Theobald, N., Choi, U., Su, L., Marquesen, M., et al. (2016). Lentiviral hematopoietic stem cell gene therapy for X-linked severe combined immunodeficiency. Sci. Transl. Med. *8*, 335ra57.

Feinberg, M.B., Baltimore, D., and Frankel, A.D. (1991). The role of Tat in the human immunodeficiency virus life cycle indicates a primary effect on transcriptional elongation. Proc. Natl. Acad. Sci. U S A *88*, 4045–4049.

Garcia, M.A., Gil, J., Ventoso, I., Guerra, S., Domingo, E., Rivas, C., and Esteban, M. (2006). Impact of protein kinase PKR in cell biology: from antiviral to antiproliferative action. Microbiol. Mol. Biol. Rev. *70*, 1032–1060.

Harris, J.W. (1950). Studies on the destruction of red blood cells. VIII. Molecular orientation in sickle cell hemoglobin solutions. Proc. Soc. Exp. Biol. Med. *75*, 197–201.

Heffner, G.C., Bonner, M., Christiansen, L., Pierciey, F.J., Campbell, D., Smurnyy, Y., Zhang, W., Hamel, A., Shaw, S., Lewis, G., et al. (2018). Prostaglandin E2 increases lentiviral vector transduction efficiency of adult human hematopoietic stem and progenitor cells. Mol. Ther. *26*, 320–328.

Hoffman, R. (2018). Hematology: Basic Principles and Practice (Elsevier).

Hu, P., Bi, Y., Ma, H., Suwanmanee, T., Zeithaml, B., Fry, N.J., Kohn, D.B., and Kafri, T. (2018). Superior lentiviral vectors designed for BSL-0 environment abolish vector mobilization. Gene Ther. *25*, 454–472.

Kanter, J., Walters, M.C., Hsieh, M., Krishnamurti, L., Kwiatkowski, J.L., Kamble, R., von Kalle, C., Joseney-Antoine, M., Pierciey, F.J., Jr., Shi, W., et al. (2017). Interim results from a phase 1/2 clinical study of lentiglobin gene therapy for severe sickle cell disease. Blood *130*, 527.

Kao, S.Y., Calman, A.F., Luciw, P.A., and Peterlin, B.M. (1987). Anti-termination of transcription within the long terminal repeat of HIV-1 by tat gene product. Nature *330*, 489–493.

Koeffler, H., Billing, R., Lusis, A., Sparkes, R., and Golde, D. (1980). An undifferentiated variant derived from the human acute myelogenous leukemia cell line (KG-1). Blood *56*, 265–273.

Kumar, M., Keller, B., Makalou, N., and Sutton, R.E. (2001). Systematic determination of the packaging limit of lentiviral vectors. Hum. Gene Ther. *12*, 1893–1905.

Laspia, M.F., Rice, A.P., and Mathews, M.B. (1989). HIV-1 Tat protein increases transcriptional initiation and stabilizes elongation. Cell *59*, 283–292.

Levasseur, D.N., Ryan, T.M., Pawlik, K.M., and Townes, T.M. (2003). Correction of a mouse model of sickle cell disease: lentiviral/antisickling $^{N}{}_{L}$-globin gene transduction of unmobilized, purified hematopoietic stem cells. Hemoglobin *102*, 4312–4319.

Lisowski, L., and Sadelain, M. (2008). Current status of globin gene therapy for the treatment of β-thalassaemia. Br. J. Haematol. *141*, 335–345.

Maetzig, T., Galla, M., Brugman, M., Loew, R., Baum, C., and Schambach, A. (2010). Mechanisms controlling titer and expression of bidirectional lentiviral and gammaretroviral vectors. Gene Ther. *17*, 400–411.

Mann, D.A., Mikaélian, I., Zemmel, R.W., Green, S.M., Lowe, A.D., Kimura, T., Singh, M., Butler, P.J., Gait, M.J., and Karn, J. (1994). A molecular rheostat: co-operative rev binding to stem I of the rev-response element modulates human immunodeficiency virus type-1 late gene expression. J. Mol. Biol. *241*, 193–207.

Masiuk, K.E., Zhang, R., Osborne, K., Hollis, R.P., Campo-Fernandez, B., and Kohn, D.B. (2019). PGE2 and poloxamer synperonic F108 enhance transduction of human HSPCs with a β-globin lentiviral vector. Mol. Ther. Methods Clin. Dev. *13*, 390–398.

Modell, B., and Darlison, M. (2008). Global epidemiology of haemoglobin disorders and derived service indicators. Bull. World Health Organ. *86*, 480–487.

Morgan, R.A., Unti, M.J., Aleshe, B., Brown, D., Osborne, K.S., Koziol, C., Ayoub, P.G., Smith, O.B., O'Brien, R., and Tam, C. (2019). Improved titer and gene transfer by lentiviral vectors using novel, small β-globin locus control region elements. Mol. Ther. https://doi.org/10.1016/j.ymthe.2019.09.020.

Morgan, R.A., Gray, D., Lomova, A., and Kohn, D.B. (2017). Hematopoietic stem cell gene therapy: progress and lessons learned. Cell Stem Cell *21*, 574–590.

Nash, G.B., Johnson, C.S., and Meiselman, H.J. (1986). Influence of oxygen tension on the viscoelastic behavior of red blood cells in sickle cell disease. Blood *67*, 110–118.

Parada, C.A. (1996). Enhanced processivity of RNA polymerase II triggered by Tat-induced phosphorylation of its carboxy-terminal. Nature *384*, 375–378.

Park, H.H., Triboulet, R., Bentler, M., Guda, S., Du, P., Xu, H., Gregory, R.I., Brendel, C., and Williams, D.A. (2018). DROSHA knockout leads to enhancement of viral titers for vectors encoding miRNA-adapted shRNAs. Mol. Ther. Nucleic Acids *12*, 591–599.

Pauling, L., Itano, H.A., Singer, S.J., and Wells, I.C. (1949). Sickle cell anemia, a molecular disease. Science *110*, 543–548.

Petrillo, C., Thorne, L.G., Unali, G., Schiroli, G., Giordano, A.M.S., Piras, F., Cuccovillo, I., Petit, S.J., Ahsan, F., Noursadeghi, M., Clare, S., et al. (2018). Cyclosporine H overcomes innate immune

restrictions to improve lentiviral transduction and gene editing in human hematopoietic stem cells. Cell Stem Cell *23*, 820–832.e9.

Pindel, A., and Sadler, A. (2011). The role of protein kinase R in the interferon response. J. Interf. Cytokine Res. *31*, 59–70.

Pollard, V.W., and Malim, M.H. (1998). The HIV-1 rev protein. Annu. Rev. Microbiol. *52*, 491–532.

Poling, B.C., Tsai, K., Kang, D., Ren, L., Kennedy, E.M., and Cullen, B.R. (2017). A lentiviral vector bearing a reverse intron demonstrates superior expression of both proteins and microRNAs. RNA Biol. *14*, 1570–1579.

Romero, Z., Urbinati, F., Geiger, S., Cooper, A.R., Wherley, J., Kaufman, M.L., Hollis, R.P., de Assin, R.R., Senadheera, S., Sahagian, A., Jin, X., et al. (2013). β-Globin gene transfer to human bone marrow for sickle cell disease. J. Clin. Invest. *123*, 3317.

Romani, B., Engelbrecht, S., and Glashoff, R.H. (2010). Functions of Tat: the versatile protein of human immunodeficiency virus type 1. J. Gen. Virol. *91*, 1–12.

Samuel, C.E. (2001). Antiviral actions of interferons. Clin. Microbiol. Rev. *14*, 778–809.

Schambach, A., Bohne, J., Chandra, S., Will, E., Margison, G.P., Williams, D.A., and Baum, C. (2006). Equal potency of gammaretroviral and lentiviral SIN vectors for expression of O^6-methylguanine-DNA methyltransferase in hematopoietic cells. Mol. Ther. *13*, 391–400.

Urbinati, F., Campo Fernandez, B., Masiuk, K.E., Poletti, V., Hollis, R.P., Koziol, C., Kaufman, M.L., Brown, D., Mavilio, F., and Kohn, D.B. (2018). Gene therapy for sickle cell disease: a lentiviral vector comparison study. Hum. Gene Ther. *29*, 1153–1166.

Zhang, Z., Klatt, A., Henderson, A.J., and Gilmour, D.S. (2007). Transcription termination factor Pcf11 limits the processivity of Pol II on an HIV provirus to repress gene expression. Genes Dev. *21*, 1609–1614.

Zufferey, R., Dull, T., Mandel, R.J., Bukovsky, A., Quiroz, D., Naldini, L., and Trono, D. (1998). Self-inactivating lentivirus vector for safe and efficient in vivo gene delivery. J. Virol. *72*, 9873–9880.

Stem Cell Reports, Volume *16*

Supplemental Information

β-Globin Lentiviral Vectors Have Reduced Titers due to Incomplete Vector RNA Genomes and Lowered Virion Production

Jiaying Han, Kevin Tam, Feiyang Ma, Curtis Tam, Bamidele Aleshe, Xiaoyan Wang, Jason P. Quintos, Marco Morselli, Matteo Pellegrini, Roger P. Hollis, and Donald B. Kohn

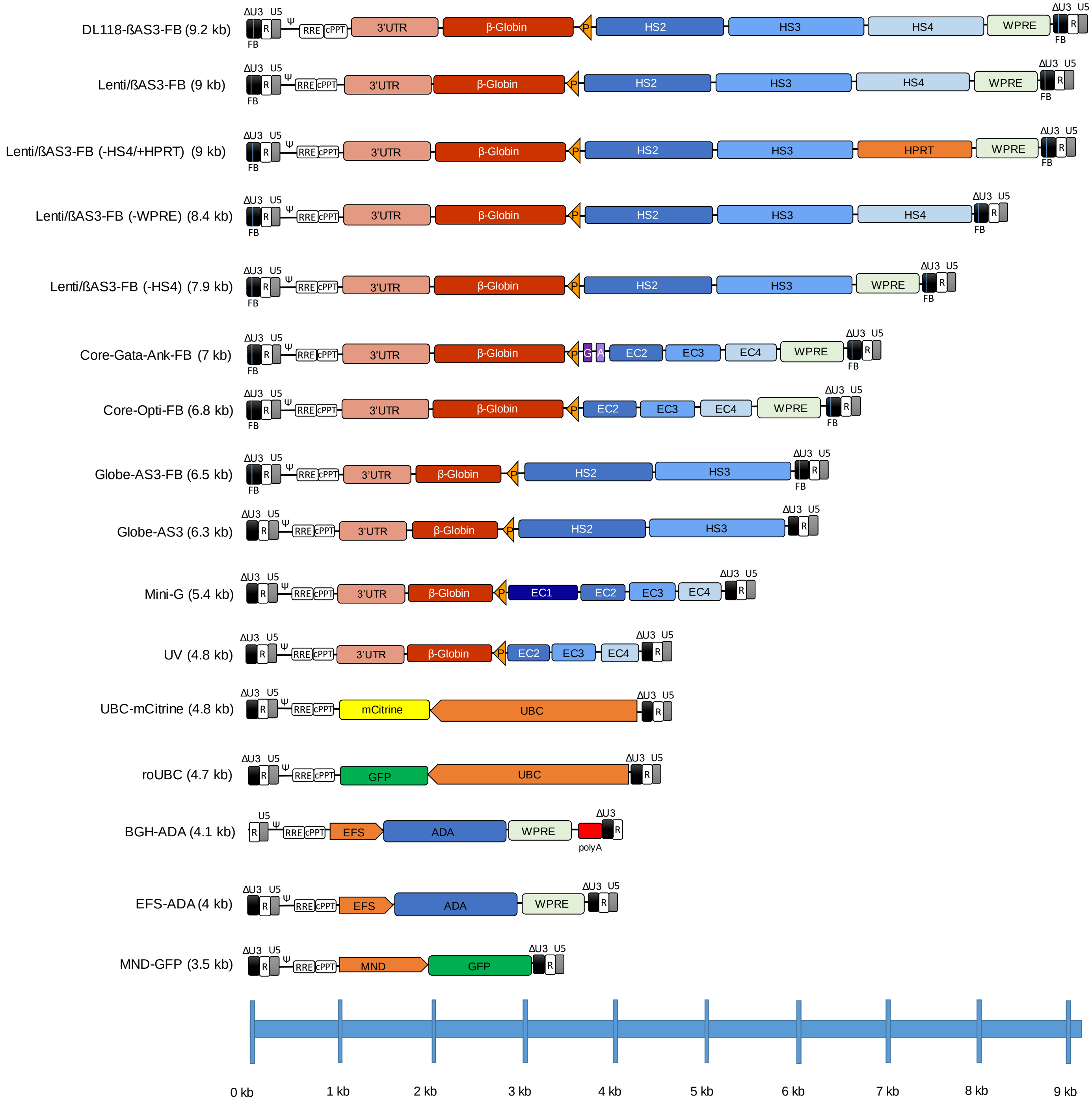
DL118-ßAS3-FB (9.2 kb)
Lenti/ßAS3-FB (9 kb)
Lenti/ßAS3-FB (-HS4/+HPRT) (9 kb)
Lenti/ßAS3-FB (-WPRE) (8.4 kb)
Lenti/ßAS3-FB (-HS4) (7.9 kb)
Core-Gata-Ank-FB (7 kb)
Core-Opti-FB (6.8 kb)
Globe-AS3-FB (6.5 kb)
Globe-AS3 (6.3 kb)
Mini-G (5.4 kb)
UV (4.8 kb)
UBC-mCitrine (4.8 kb)
roUBC (4.7 kb)
BGH-ADA (4.1 kb)
EFS-ADA (4 kb)
MND-GFP (3.5 kb)
ΔU3
U5
R
FB
Ψ
RRE
cPPT
3'UTR
β-Globin
P
HS2
HS3
HS4
HPRT
WPRE
G
A
EC1
EC2
EC3
EC4
mCitrine
UBC
GFP
EFS
ADA
polyA
MND
0 kb
1 kb
2 kb
3 kb
4 kb
5 kb
6 kb
7 kb
8 kb
9 kb

Figure S1. Maps of the lentiviral vector proviruses. All vectors consist of a pCCL backbone and differ only by the internal promoters and transgene cassettes. ΔU3, R, U5 are long terminal repeats (LTR). FB, FII-BEAD insulator. Ψ, packaging signal. RRE, Rev response element. cPPT, central polypurine tract. 3' UTR, ß-globin gene 3' UTR enhancer elements. P, promoter. G, Gata1 erythroid transcription factor binding site. A, ankyrin-1 barrier insulator element. HS2, HS3, HS4, ß-globin locus hypersensitive sites 2, 3 and 4. EC2, EC3, EC4, enhancer core elements. WPRE, mutated woodchuck hepatitis virus post-transcriptional regulatory element. ß-Globin, ß-Globin gene cassette. UBC, ubiquitin C promoter. EFS, elongation factor 1 alpha promoter. ADA, codon-optimized adenosine deaminase cDNA. BGH-ADA is shown in its RNA form because it is a truncated vector that cannot integrate.

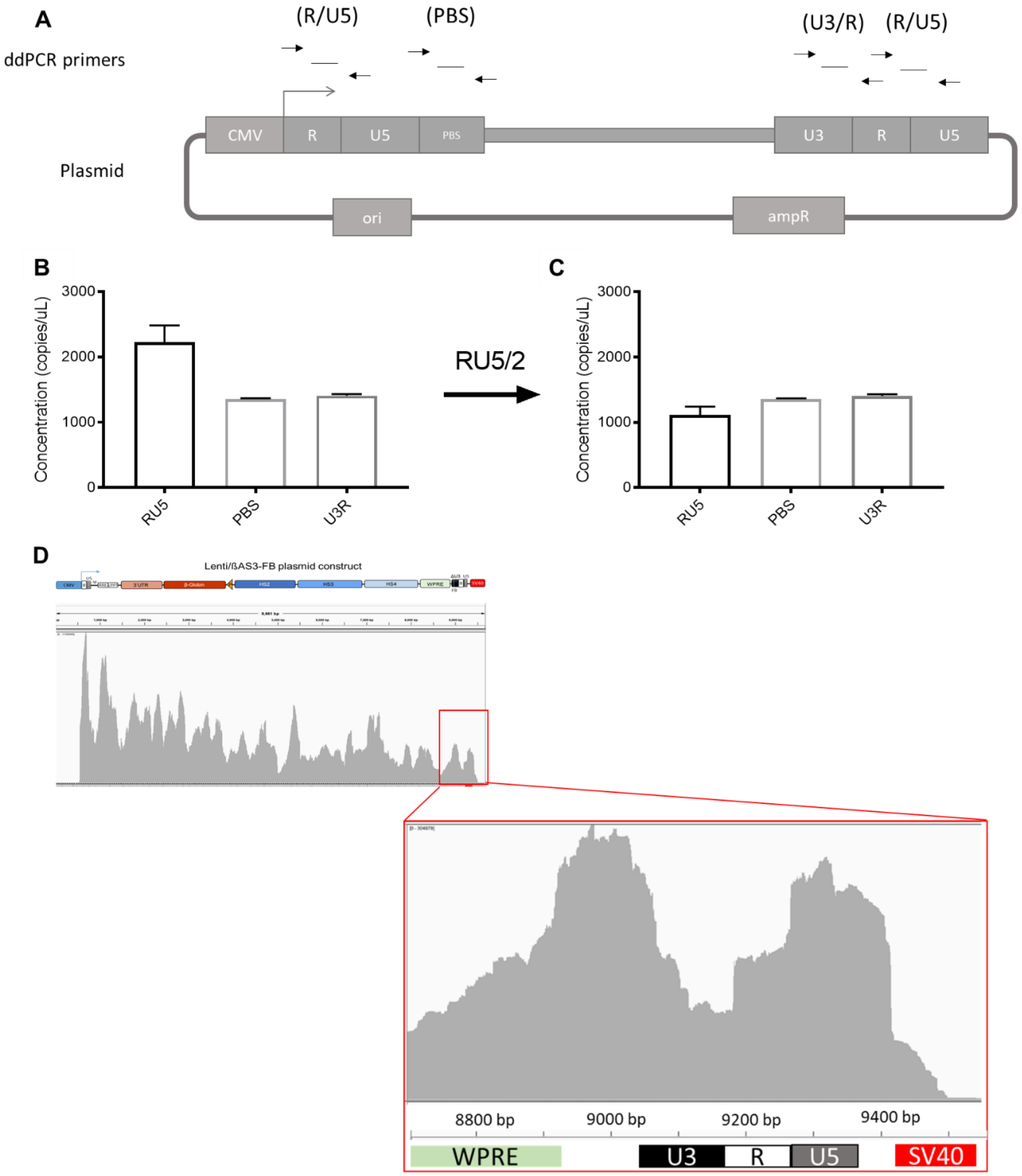
A
ddPCR primers
(R/U5)
(PBS)
(U3/R)
(R/U5)
Plasmid
CMV
R
U5
PBS
U3
R
U5
ori
ampR
B
Concentration (copies/uL)
3000
2000
1000
0
RU5
PBS
U3R
RU5/2
C
Concentration (copies/uL)
3000
2000
1000
0
RU5
PBS
U3R
D
Lenti/ßAS3-FB plasmid construct
8800 bp
9000 bp
9200 bp
9400 bp
WPRE
U3
R
U5
SV40

Figure S2. Quantification of ddPCR primer amplification efficiencies and viral RNA transcription readthrough. (A) Schematic representation of primer binding sites in vector plasmids. (B) Copy number/ul by R/U5, PBS and U3/R primers and probes in Lenti/ßAS3-FB plasmids (n=2 independent experiments). (C) Normalized copy number/ul. R/U5 copy number was divided by two, because plasmids contain two copies of R/U5 (bars represent mean with SEM; n=2 independent experiments). (D) Alignment of RNA-seq reads to 3'LTR to SV40 polyadenylation sites in Integrative Genomics Viewers.

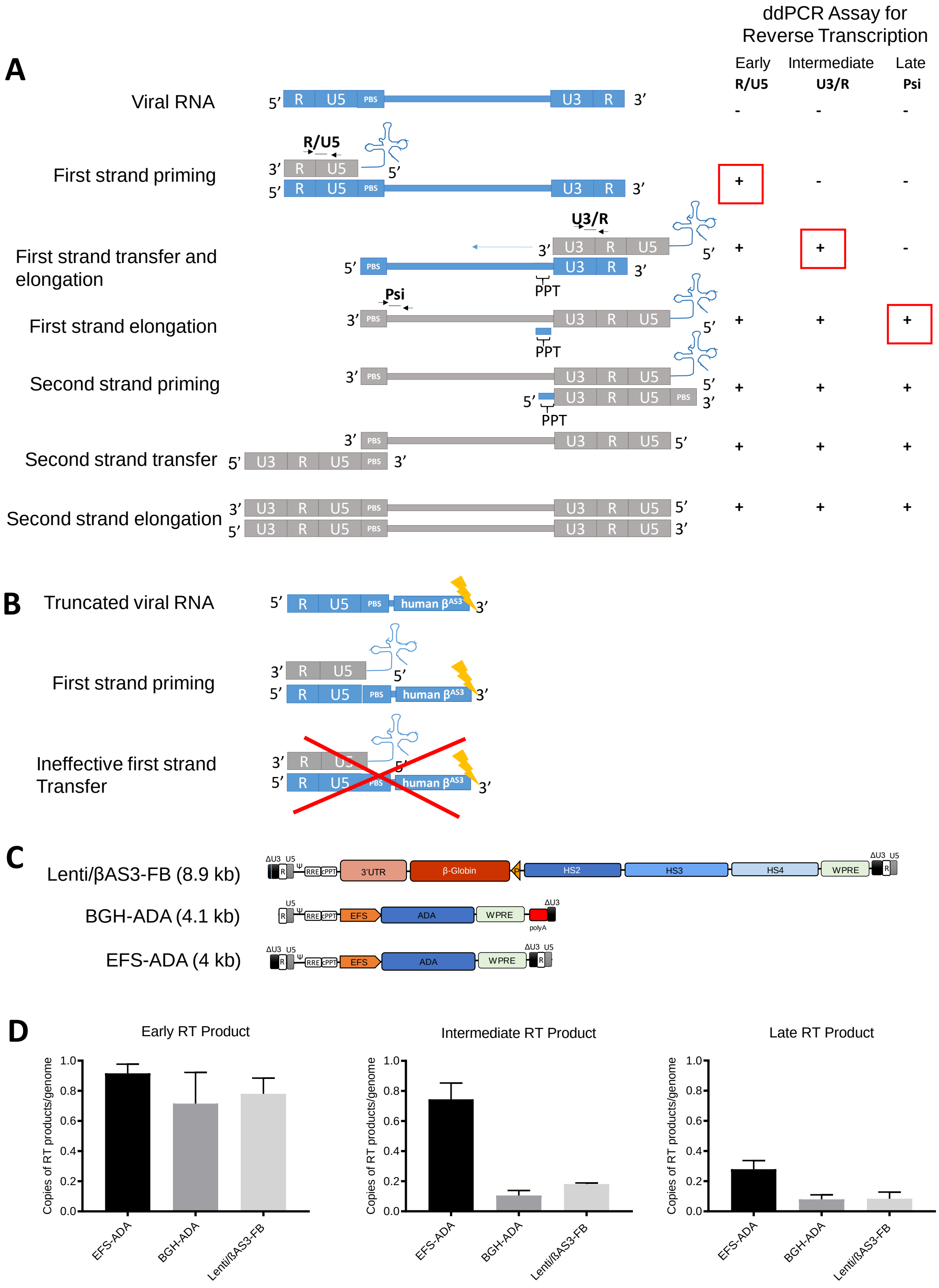

A
B
C
D
ddPCR Assay for Reverse Transcription
Early
R/U5
Intermediate
U3/R
Late
Psi
Viral RNA
First strand priming
First strand transfer and elongation
First strand elongation
Second strand priming
Second strand transfer
Second strand elongation
PPT
PBS
Truncated viral RNA
First strand priming
Ineffective first strand Transfer
human βAS3
Lenti/βAS3-FB (8.9 kb)
BGH-ADA (4.1 kb)
EFS-ADA (4 kb)
3'UTR
β-Globin
HS2
HS3
HS4
WPRE
EFS
ADA
polyA
RRE
cPPT
Early RT Product
Intermediate RT Product
Late RT Product
Copies of RT products/genome
EFS-ADA
BGH-ADA
Lenti/βAS3-FB

Figure S3. Lenti/ßAS3-FB failed reverse transcription at the first strand transfer step. (A) Schematic representation of the mechanism of reverse transcription (RT) and the ddPCR assay used to quantify reverse transcribed products. The ddPCR primers and probes were designed to quantify different stages of RT when RT products become detectable. The R/U5 primers quantify initial reverse transcribed products, the U3/R primers quantify intermediate reverse transcribed products, and the Psi primers quantify late reverse transcribed products. Blue box: viral RNA. Gray box: viral DNA. (B) Proposed mechanism of truncated RNA failing reverse transcription at the first strand transfer step. (C) Schematic representation of the vector RNA of EFSADA and the truncated version BGHADA. (D) Quantification of reverse transcribed products of Lenti/ßAS3-FB, EFS-ADA and BGH-ADA LVs in KG1a cells (bars represent mean with SEM; n=5 dishes of identical cultures from 2 independent experiments). BGH-ADA is a truncated form of EFSADA LV with the Bovine Growth Hormone (BGH) polyadenylation signal between WPRE and 3'LTR. KG1a cells were transduced with Lenti/ßAS3-FB, EFS-ADA, or BGH-ADA LVs at equal levels of Initial RNA in the presence of PGE2, poloxamer, and benzonase. Cells were harvested at 24 h for gDNA extraction. Reverse transcription products were quantified by ddPCR with R/U5, U3/R, Psi, and SDC4 primers.

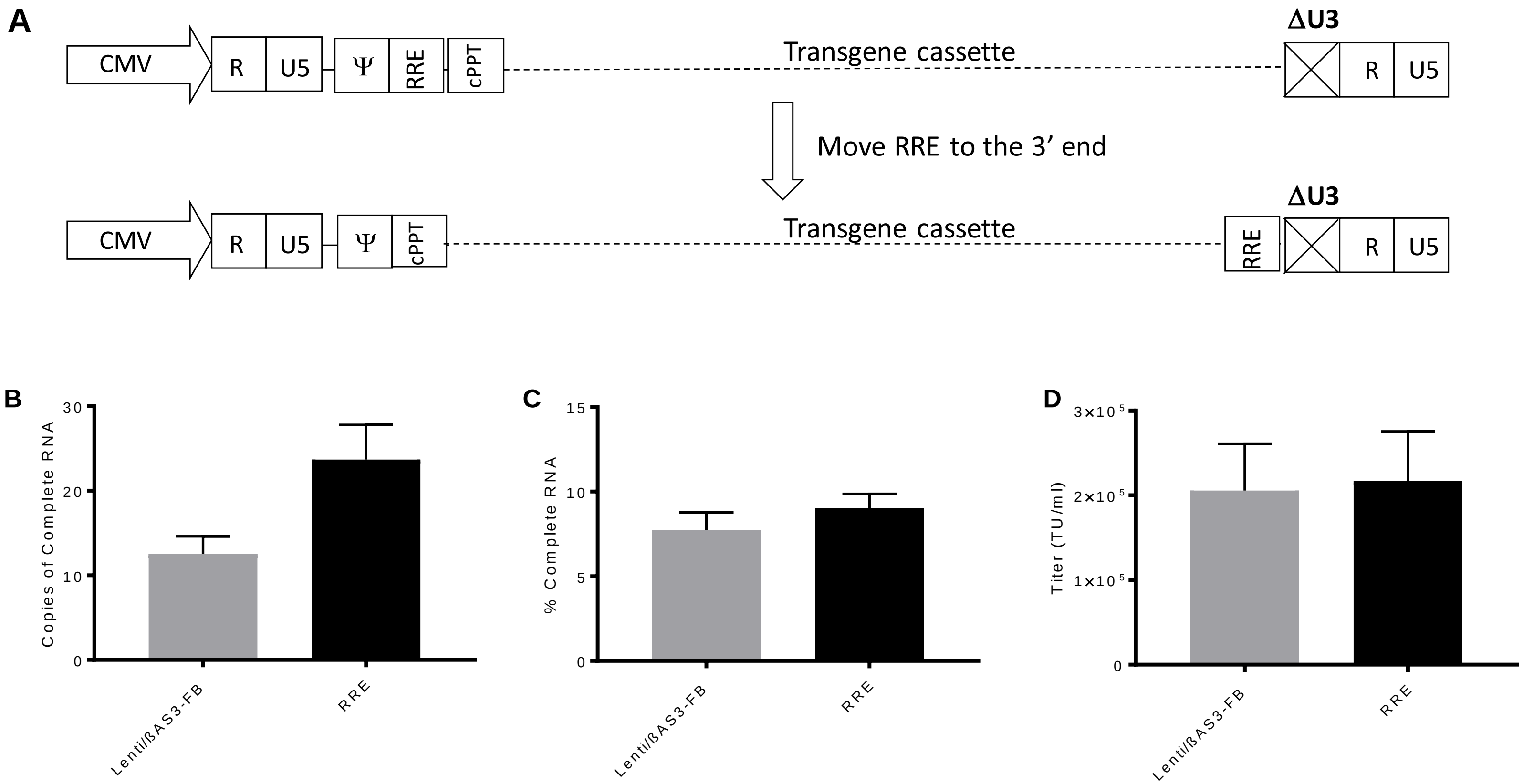
A
CMV
R
U5
Ψ
RRE
cPPT
Transgene cassette
ΔU3
R
U5
Move RRE to the 3’ end
CMV
R
U5
Ψ
cPPT
Transgene cassette
RRE
ΔU3
R
U5
B
Copies of Complete RNA
30
20
10
0
Lenti/ßAS3-FB
RRE
C
% Complete RNA
15
10
5
0
Lenti/ßAS3-FB
RRE
D
Titer (TU/ml)
3×10⁵
2×10⁵
1×10⁵
0
Lenti/ßAS3-FB
RRE

Figure S4. Moving the RRE element to the 3’ LTR did not increase viral titer. (A) Schematic representation of the vector constructs. The RRE element in Lenti/ßAS3-FB was moved from a site downstream of the 5’ LTR to a site upstream of the 3’ LTR to generate a new vector termed as Lenti/ßAS3-FB-RRE. (B) Absolute quantification and (C) percentage of viral RNA from unconcentrated viral supernatant of Lenti/ßAS3-FB-RRE and Lenti/ßAS3-FB measured by ddPCR with R/U5 and U3/R primers and probes (n=3 dishes of identical cultures treated and analyzed in two independent experiments). (D) Viral titers of Lenti/ßAS3-FB-RRE and Lenti/ßAS3-FB LVs (n=3 dishes of identical cultures treated and analyzed in two independent experiments; bars represent mean with SEM).

.

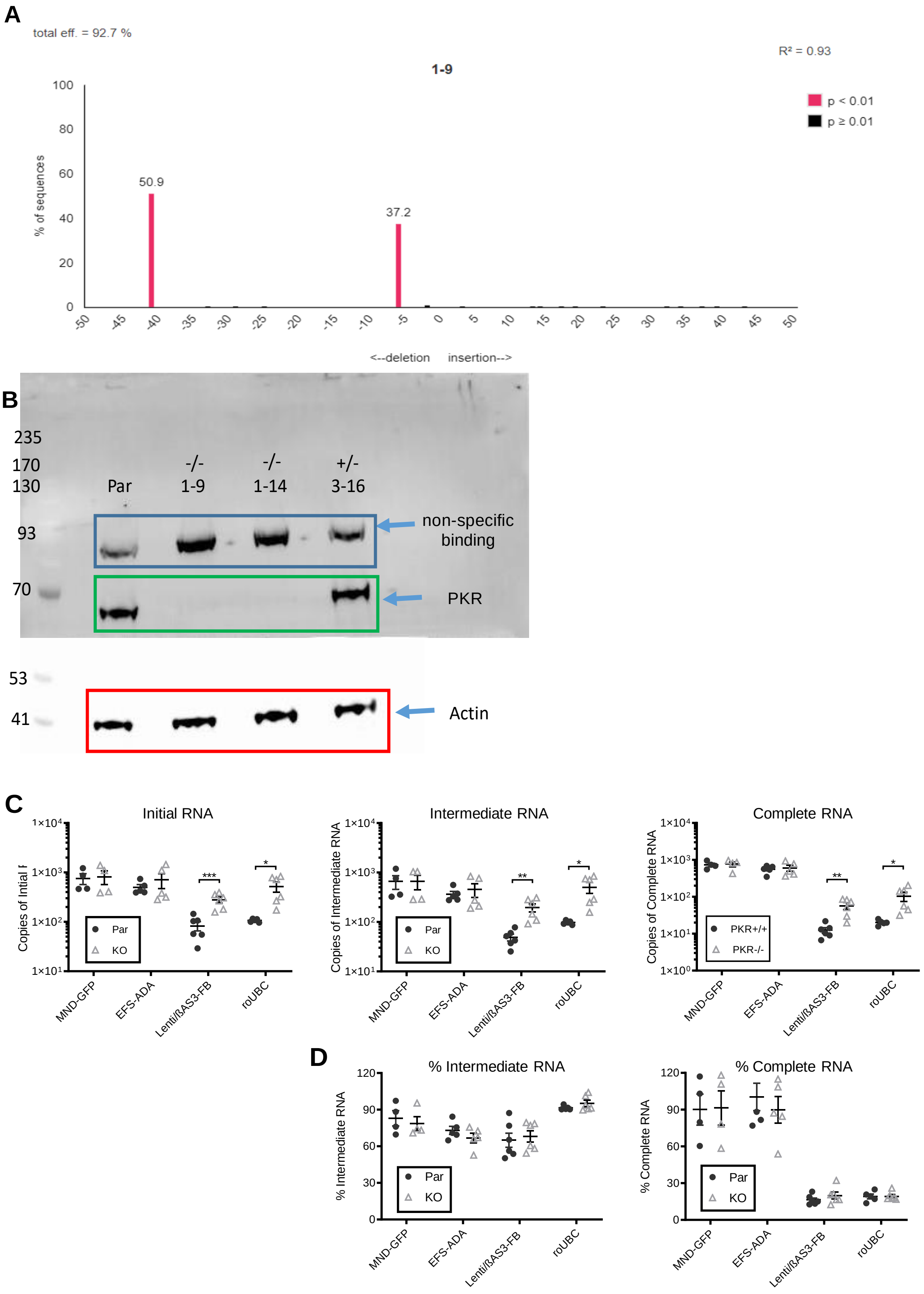
A
total eff. = 92.7 %
R² = 0.93
1-9
p < 0.01
p ≥ 0.01
% of sequences
50.9
37.2
<--deletion insertion-->
B
235
170
130
93
70
53
41
Par
-/-
1-9
-/-
1-14
+/-
3-16
non-specific binding
PKR
Actin
C
Initial RNA
Intermediate RNA
Complete RNA
Copies of Intermediate RNA
Copies of Complete RNA
Par
KO
PKR+/+
PKR-/-
MND-GFP
EFS-ADA
Lenti/βAS3-FB
roUBC
D
% Intermediate RNA
% Complete RNA
Par
KO

Figure S5. Knocking out *PKR* increased the level of all three RNA species in reverse-oriented vectors without changing the RNA completeness. (A) Assessment of genome disruption by TIDE sequencing of the *PKR-/-* isogenic 293T cell line. Cas9 and guide RNA were introduced to the cells by lipofection of PX330 plasmids bearing the Cas9 and guide RNA sequence targeting PKR. Cells were sorted for single cell clones, and the isogenic cell clones were harvested three weeks later for genomic DNA extraction and Tide sequencing analysis. (B) PKR protein expression in different isogenic cell clones measured by Western blot. Clones 1-9 and 1-14 were homozygous *PKR-/-* cell clones, and 3-16 was a heterozygous *PKR+/-* cell clone by TIDE sequencing. Parental (Par) 293T cell lysate was used as a positive control. ß-actin was used as the loading control. The expected size of PKR protein was 74 kDa. (C) Absolute quantification of Initial (R/U5), Intermediate (PBS), and Complete (U3/R) RNA in vector particles by ddPCR (bars represent mean with SEM; n=4-6 dishes of identical cultures treated and analyzed in 3 independent experiments; unpaired t test, *$p<0.05$, **$p<0.01$, ***$p<0.001$). (D) Percentage Intermediate and Complete RNA in vector particles measured by ddPCR (bars represent mean with SEM; n=4-6 dishes of identical cultures from 3 independent experiments; unpaired t test, *$p<0.05$, **$p<0.01$, ***$p<0.001$). RNA was extracted from vector particles, treated with DNAse, reverse transcribed with random primers, and quantified by ddPCR.

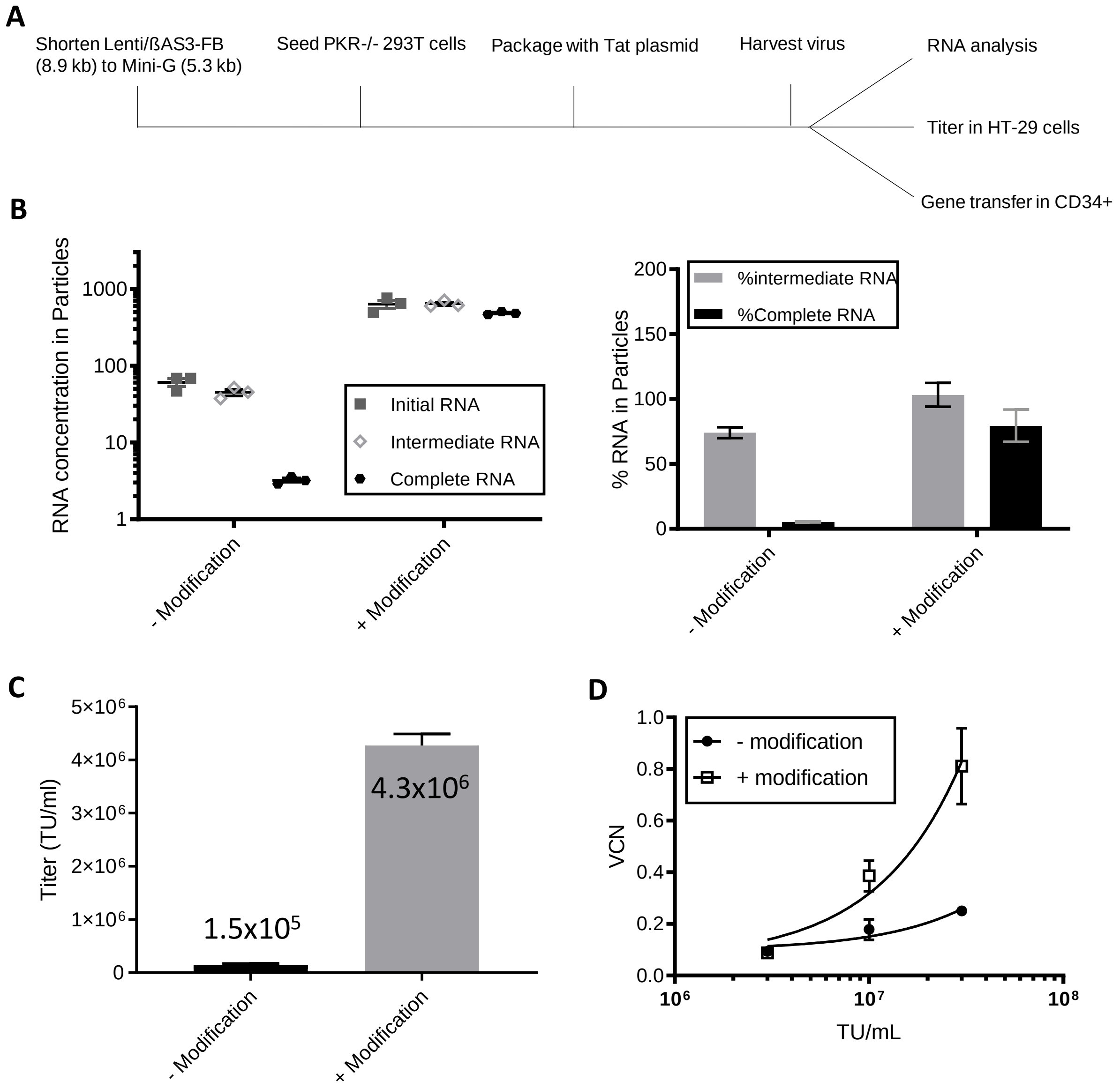
A
Shorten Lenti/ßAS3-FB
(8.9 kb) to Mini-G (5.3 kb)
Seed PKR-/- 293T cells
Package with Tat plasmid
Harvest virus
RNA analysis
Titer in HT-29 cells
Gene transfer in CD34+
B
RNA concentration in Particles
1000
100
10
1
Initial RNA
Intermediate RNA
Complete RNA
- Modification
+ Modification
% RNA in Particles
200
150
100
50
0
%intermediate RNA
%Complete RNA
- Modification
+ Modification
C
Titer (TU/ml)
5×10^6
4×10^6
3×10^6
2×10^6
1×10^6
0
1.5x10^5
4.3x10^6
- Modification
+ Modification
D
VCN
1.0
0.8
0.6
0.4
0.2
0.0
- modification
+ modification
10^6
10^7
10^8
TU/mL

Figure S6. Combining the three modifications for the production of ß-globin vectors increased viral RNA, vector titer and CD34+ cell infectivity. (A) The schematic representation of the three modifications: shortening the vector length, packaging with TAT plasmids and packaging in PKR-/- cells. (B) The absolute quantification and percentage of viral RNA in viral particles measured by ddPCR. (C) Titers of unconcentrated ß-globin vectors +/- 3 modifications (bars represent mean with SEM; n=3 dishes of identical cultures treated and analyzed in 2 independent experiments). (D) Infectivity of ß-globin vectors +/- 3 modifications in BM CD34+ HSPCs (bars represent mean with SEM; n=3 independent experiments; linear regression, comparison of the slopes, p=0.0009). Data represent measurements from three independent human BM CD34+ HSPCs donors.

Table S1. Sequences of primer and probes

**Name**	**Sequence 5'→3'**
R/U5 FWD	GCTAACTAGGGAACCCACTGCT
R/U5 REV	GGGTCTGAGGGATCTCTAGTTACCA
R/U5 Probe	FAM- CTTCAAGTAGTGTGTGCCCGTCTGT-31ABFQ
PBS FWD	AAGTAGTGTGTGCCCGTCTG
PBS REV	CCTCTGGTTTCCCTTTCGCT
PBS Probe	FAM-CCCTCAGACCCTTTTAGTCAGTGTGGAAAATCTCTAG-31ABFQ
U3/R FWD	AGCAGTGGGTTCCCTAGTTAG
U3/R REV	GGGACTGGAAGGGCTAATTC
U3/R Probe	FAM-AGAGACCCAGTACAAGCAAAAAGCAG-31ABFQ
Psi FWD	CTTGAAAGCGAAAGGGAAACC
Psi REV	CGCACCCATCTCTCTCCTTCT
Psi Probe	FAM-AGCTCTCTCGACGCAGGACTCGGC-31ABFQ
SDC4 FWD	CAGGGTCTGGGAGCCAAGT
SDC4 REV	GCACAGTGCTGGACATTGACA
SDC4 Probe	FAM - CCCACCGAACCCAAGAAACTAGAGGAGAAT - TAMRA

Supplemental Methods

Reverse Transcription Assay

Raw viral supernatants of EFS-ADA, Lenti/βAS3-FB and BGH-ADA vectors with the same amounts of Initial RNA were used to transduce KG1a cells. Benzonase, 50 U/mL (MilliporeSigma, Burlington, MA) and transduction enhancers, Poloxamer 1 mg/mL (Kolliphor P338; BSAF, Ludwigshafen, Germany) and PGE2 10 µM (Cayman Chemicals, Ann Arbor, MI, USA) were added to the cells at the same time as the addition of LV. The preparation of transduction enhancers was previously described in Masiuk et al (Masiuk et al., 2019). Cells were harvested at 24 hours after transduction, and DNA was extracted using Qiagen Blood and Cell culture DNA Mini Kit (Qiagen).

The copies of reverse-transcribed DNA products were measured by ddPCR, calculated as the ratio of the copies of the viral genomes to the copies of the *SDC4* endogenous reference gene. Different PCR primers were used to quantify early, intermediate and late reverse transcribed products. The R/U5 primers and probe were used to quantify early reverse transcribed products, and the U3/R primer and probe were used to quantify intermediate reverse transcribed products, as described earlier. The cycling conditions for ddPCR consisted of 95°C for 10 min for one cycle, (94°C for 30 s and 60°C for 1 min) for 40 cycles, 10 min at 98°C for one cycle, and a 12°C hold.

Generation of *PKR-/-* 293T Cell Line

Single guide RNA (sgRNA) targeting *PKR* exon 1 were designed using Benchling CRISPR online tool, and paired oligonucleotides were ordered from Integrated DNA Technologies (San Diego, CA). Paired oligonucleotides encoding the sgRNA were annealed and cloned into the PX330-U6-Chimeric_BB-CBh-hSpCas9 expression vector (#42230, Addgene, Watertown, Massachusetts) as previously described (Hsu et al., 2013; Cong et al., 2013). Maxi-prepped expression plasmids were filtered through a 0.22 µM filter and introduced to 293T cells by transient transfection with 5 µg of plasmids, 1.5 mL OPTI-MEN (ThermoFisher, Waltham, MA) and 35 uL TransIT-293 (Mirus Bio) in a 10 cm plate, following the manufacturer’s protocol. Cell sorting to isolate single cells was conducted 48 hours after transfection at The UCLA Broad Stem Cell

Research Center Flow Cytometry Core. After three weeks of culture, isogenic cell clones were collected for genomic DNA extraction, PCR amplification, Tide sequencing analysis, and Western blot analysis for PKR protein expression, as previously described in Hu et al to isolate *PKR*-/- clones with bi-allelic *PKR* knock-out and no detectable residual PKR expression (Birkman et al., 2014; Hu et al, 2018),. One *PKR* -/- clone was expanded and used for LV packaging.

LV Production & Titration

LVs were packaged by transient transfection of 293T cells with fixed amounts of HIV Gag/Pol, Rev and VSV-G expression plasmids and equimolar amounts of each of the different vector transfer plasmids using TransIT-293 (Mirus Bio, Madison, WI). Approximately 20 hours after transfection, the culture medium was changed to D10 containing 10 mM sodium butyrate and 20 mM HEPES. After 6-8 hours, cells were washed with PBS and cultured in fresh D10. Viral supernatants were collected ~40 hours after sodium butyrate induction and filtered through a 0.45-μm filter. Viral supernatants were processed and concentrated by ultracentrifugation at 26,000 rpm for 90 minutes, when necessary. Both unconcentrated and concentrated viral supernatant were harvested and stored at -80°C.

Viral supernatant was titrated in HT29 cells as described in the method section. VCN was determined as the ratio of the copies of the HIV-1 PSI region to the copies of the *SDC4* endogenous reference gene. Droplet generation was performed as described in Hindson et al (Hindson et al., 2011). The droplet emulsion was then transferred to a 96-well polypropylene plate (Eppendorf, Hamburg, Germany) and amplified in a conventional thermal cycler (T100; Bio-Rad). Thermal cycling conditions consisted of 95°C for 10 min for one cycle, (94°C for 30 s and 60°C for 1 min) for 55 cycles, 10 min at 98°C for one cycle, and a 12°C hold. After PCR, the 96-well plate was transferred to a ddPCR droplet reader (Bio-Rad) for data acquisition and analysis performed with the QuantaSoft software (Bio-Rad).

Supplemental References

Brinkman, E. K., Chen, T., Amendola, M. & Steensel, B. Van. Easy quantitative assessment of genome editing by sequence trace decomposition. 42, (2014).

Hindson, B. J. *et al.* High-Throughput Droplet Digital PCR System for Absolute Quantitation of DNA Copy Number. 8604–8610 (2011).

Hsu, P. D. *et al.* DNA targeting specificity of RNA-guided Cas9 nucleases. 31, (2013).

Cong, L. *et al.* Multiplex genome engineering using CRISPR/Cas systems. *Science.* 339, 819–823 (2013).

Hu, P. *et al.* Superior lentiviral vectors designed for BSL-0 environment abolish vector mobilization. *Gene Ther.* 454–472 (2018). doi:10.1038/s41434-018-0039-2

Masiuk, K. E. *et al.* PGE2 and Poloxamer Synperonic F108 Enhance Transduction of Human HSPCs with a β-Globin Lentiviral Vector. *Mol. Ther. Methods Clin. Dev.* 13, 390–398 (2019).
